# Sweat the Fall Stuff: Physical Activity Moderates the Association of White Matter Hyperintensities With Falls Risk in Older Adults

**DOI:** 10.3389/fnhum.2021.671464

**Published:** 2021-05-21

**Authors:** Rachel A. Crockett, Ryan. S. Falck, Elizabeth Dao, Chun Liang Hsu, Roger Tam, Walid Alkeridy, Teresa Liu-Ambrose

**Affiliations:** ^1^Aging, Mobility, and Cognitive Neuroscience Laboratory, The University of British Columbia, Vancouver, BC, Canada; ^2^Djavad Mowafaghian Centre for Brain Health, The University of British Columbia, Vancouver, BC, Canada; ^3^Centre for Hip Health and Mobility, Vancouver Coastal Health Research Institute, Vancouver, BC, Canada; ^4^Department of Radiology, The University of British Columbia, Vancouver, BC, Canada; ^5^Hinda and Arthur Marcus Institute for Aging Research, Hebrew SeniorLife, Boston, MA, United States; ^6^Harvard Medical School, Harvard University, Boston, MA, United States; ^7^School of Biomedical Engineering, The University of British Columbia, Vancouver, BC, Canada; ^8^Division of Geriatrics, Department of Medicine, The University of British Columbia, Vancouver, BC, Canada; ^9^College of Medicine, King Saud University, Riyadh, Saudi Arabia

**Keywords:** white matter hyperintensities, physical activity, falls risk, aging, cerebrovascular disease

## Abstract

**Background:** Falls in older adults are a major public health problem. White matter hyperintensities (WMHs) are highly prevalent in older adults and are a risk factor for falls. In the absence of a cure for WMHs, identifying potential strategies to counteract the risk of WMHs on falls are of great importance. Physical activity (PA) is a promising countermeasure to reduce both WMHs and falls risk. However, no study has yet investigated whether PA attenuates the association of WMHs with falls risk. We hypothesized that PA moderates the association between WMHs and falls risk.

**Methods:** Seventy-six community-dwelling older adults aged 70–80 years old were included in this cross-sectional study. We indexed PA using the Physical Activity Score for the Elderly (PASE) Questionnaire. Falls risk was assessed using the Physiological Profile Assessment (PPA), and WMH volume (mm^3^) was determined by an experienced radiologist on T2-weighted and PD-weighted MRI scans. We first examined the independent associations of WMH volume and PASE score with PPA. Subsequently, we examined whether PASE moderated the relationship between WMH volume and PPA. We plotted simple slopes to interpret the interaction effects. Age, sex, and Montreal Cognitive Assessment (MoCA) score were included as covariates in all models.

**Results:** Participants had a mean age of 74 years (SD = 3 years) and 54 (74%) were female. Forty-nine participants (66%) had a Fazekas score of 1, 19 (26%) had a score of 2, and 6 (8%) a score of 3. Both PASE (β = −0.26 ± 0.11; *p* = 0.022) and WMH volume (β = 0.23 ± 0.11; *p* = 0.043) were each independently associated with PPA score. The interaction model indicated that PASE score moderated the association between WMH volume and PPA (β = −0.27 ± 0.12; *p* = 0.030), whereby higher PASE score attenuated the association between WMHs and falls risk.

**Conclusion:** PA is an important moderator of falls risk. Importantly, older adults with WMH can reduce their risk of falls by increasing their PA.

## Introduction

Falls are a major global public health problem (World Health Organization, 2008; Ageing and Life Course Unit), with over a third of older adults experiencing at least one fall each year (Tinetti et al., 1994). White matter hyperintensities (WMH) are a prominent feature of cerebrovascular disease and are prevalent in older adults (de Leeuw et al., 2001). Importantly, WMHs are a major risk factor for falls (Zheng et al., 2011). As there is currently no cure for WMHs (Alber et al., 2019), strategies which can mitigate the risks of WMH on falls are needed. Physical activity (PA) is an important modifiable lifestyle factor associated with both lower WMH volumes (Burzynska et al., 2014; Dao et al., 2018; Alber et al., 2019) and reduced risk of falling (Khan et al., 2001). However, whether PA moderates the burden of WMH volume on falls risk is not well established.

White matter hyperintensities are evident in over 90% of older adults (de Leeuw et al., 2001). They are small lesions that can be identified as bright, hyperintense regions on T2-weighted, proton density-weighted, and fluid attenuated inversion recovery magnetic resonance imaging (Wardlaw et al., 2015). They are most commonly caused by damage to the connecting blood vessels, resulting in reduced blood flow and oxygen to the brain cells (Sharma et al., 2020). Damage to brain white matter in the form of WMHs may lead to a reduction in the number and quality of important neural connections (Langen et al., 2017).

A meta-analysis by Kloppenborg et al. (2014), identified both cross-sectional and longitudinal associations between WMHs and multiple cognitive domains, including attention, processing speed, and executive functions. Deficits in these cognitive domains were identified as significant predictors of future falls risk in older adults (Mirelman et al., 2012). Further, impaired gait speed, balance and functional mobility are also associated with WMHs (Zheng et al., 2011) and have been identified as significant risk factors for falls (Lord et al., 1994; Ganz et al., 2007; Quach et al., 2011). Consequently, WMHs are associated with deficits in cognition and mobility, both of which contribute to an increased risk of falling. Several studies support the notion that older adults with a history of falls are more likely to have WMHs than non-fallers (Callisaya et al., 2015; Torres et al., 2015). In addition, Srikanth et al. (2009) identified that older adults in the top quintile for total WMH volume had double the risk of falling compared with those in the bottom quintile.

Lifestyle factors, such as PA, are a possible avenue to prevent and/or reverse WMH progression (Torres et al., 2015; Dao et al., 2018). PA is defined as any bodily movement produced by skeletal muscles that requires energy expenditure (Caspersen et al., 1985). Older adults with greater WMH volume are more likely to be inactive (Saczynski et al., 2008), and lower levels of PA are predictive of greater progression of WMH volume 3 years later (Gow et al., 2012). Twelve months of PA in the form of exercise training reduces WMH volumes (Bolandzadeh et al., 2015; Suo et al., 2016) indicating that increasing PA may help prevent WMH progression.

Being more physically active is also associated with reduced falls risk. Klenk et al. (2015) showed that older adults who walked for less than 1 h per day experienced more falls compared with their more active peers. Importantly, the most successful falls prevention programs center on increasing PA (Khan et al., 2001). A meta-analysis of 116 randomized controlled trials found that PA in the form of exercise training reduced the rate of falls by 23–42% (Sherrington et al., 2020).

While PA may thus counteract WMH progression and reduces fall risk, it is still unclear whether PA moderates the association of WMHs with falls risk in older adults. Identifying whether PA reduces the association of WMH with falls risk will provide important insight for the development of falls prevention programs targeting this population. The aim of this study is to determine whether level of PA moderates the association between WMH volume and falls risk. It is hypothesized that greater levels of PA will attenuate the association of WMH with falls risk.

## Materials and Methods

### Participants

This study was a secondary cross-sectional analysis of a longitudinal study (*N* = 149) aimed at investigating the relationship between mobility, functional connectivity, and cognition (Hsu et al., 2014). For the present analysis, we only included a subset of participants (*n* = 76) with WMH on baseline MRI.

We recruited community dwelling older adults from Greater Vancouver. Participants were: (1) aged 70 to 80 years old; (2) scored >24/30 on the Mini-Mental State Examination (MMSE) (Folstein et al., 1975); (3) right hand dominant as measured by the Edinburgh Handedness Inventory (Oldfield, 1971); (4) living independently in their own homes; (5) had a visual acuity of at least 20/40, with or without corrective lenses; and (6) provided informed consent. Ethics approval (H07-00160) was obtained from the Vancouver Coastal Health Research Institute and University of British Columbia’s Clinical Research Ethics Board.

### Measures

For descriptive purposes, age in years, height in centimeters, and weight in kilograms were measured. General cognition was also assessed using the Montreal Cognitive Assessment (MoCA) (Nasreddine et al., 2005) tool.

#### Falls Risk

Falls risk was assessed using the Physiological Profile Assessment© (PPA) [Prince of Wales Medical Research Institute, Randwick, Sydney, NSW, Australia (Lord et al., 2003)]. The PPA is a validated measure for quantifying falls risk in older adults (Lord et al., 2003) with a 75% predictive accuracy for falls (Lord et al., 1994). This five component assessment battery creates a composite falls risk score from: (1) hand reaction time; (2) knee extension strength; (3) visual contrast sensitivity; (4) balance; and (5) proprioception. A higher PPA score is indicative of greater falls risk.

#### Physical Activity

The Physical Activity Scale for the Elderly (PASE) is a questionnaire designed to determine PA levels in older adults over the age of 65 (Washburn et al., 1993). The PASE combines information from household, leisure, and occupational activities typical of this population. It includes measures of frequency, duration, and level of intensity of the activity. Scores range from 0 to 793 with higher scores indicative of greater PA levels.

#### White Matter Hyperintensity Quantification

Magnetic resonance imaging (MRI) scans were conducted at the UBC MRI Research Center on a 3T Philips Achieva Scanner with an 8-channel SENSE neurovascular coil. T2-weighted and proton density (PD)-weighted structural MRI scans were acquired for each subject. The T2-weighted scan used a repetition time (TR) of 2,500 ms, echo time (TE) of 382 ms, and a 312 × 312 acquisition matrix. The PD-weighted scan, used a 3,000 ms TR, 30 ms TE, and 252 × 250 acquisition matrix.

The MRI scans were preprocessed using standard neuroimaging tools including: (1) a structure-preserving noise removal filter (SUSAN) (Smith and Brady, 1997); (2) a brain extraction tool (BET) to remove all non-brain tissue (Smith, 2002); and (3) a multiscale version of the non-parametric non-uniform intensity normalization method (N3) for MR intensity inhomogeneity correction (Sled et al., 1998). White matter hyperintensities were determined by an experienced radiologist (McAusland et al., 2010). The seeding procedure guidelines were to: (1) mark all distinct WMHs regardless of size; (2) use additional points if more than one point would help define the extent of the lesion; (3) place at least one point near the center of each lesion (McAusland et al., 2010). The WMHs were then automatically segmented by computing the extent of each lesion using a customized Parzen windows classifier (McAusland et al., 2010). The average total WMH volume across both hemispheres was used to calculate a single WMH volume value per participant (Liu-Ambrose et al., 2006).

### Data Analysis

All statistical analyses were completed in R version 4.0.3 (see Supplementary Materials 1, 2). Descriptive statistics for all participants were calculated using *tableone 0.12.0*. We conducted a linear regression model, which examined: (1) the association of WMH volume with falls risk independent of PA; and (2) the association of PA and falls risk independent of WMH volume. WMH volume and PASE score were included in the linear regression model as the independent variables of interest; PPA score was the dependent variable. Baseline age, sex and baseline MoCA score were included as covariates of no interest.

To examine whether PA moderated the association of WMH volume with falls risk, we performed a second linear regression model, which included as independent variables: (1) WMH volume, (2) PASE score, and (3) the interaction of WMH volume with PASE score. PPA score was the dependent variable of interest; age, sex, and MoCA were included as covariates. The interaction (i.e., WMH volume X PASE score) was then decomposed using model-based estimates of simple slopes, in which the relation between WMH volume and PPA score was estimated separately for low PASE score (i.e., 1 SD below the mean; ∼67/703) and high PASE score (1 SD above the mean; ∼203/793). Standardized beta estimates, standard errors, and *p-*values are presented. All significant relationships were plotted with *ggplot2 3.3.2.*

In the event that PA was found to significantly moderate the association between WMH and PPA. We conducted exploratory analyses to identify if PA moderated the association between WMH and any of the five individual components of the PPA specifically.

In accordance with our ethical guidelines, data is available upon review of specific requests.

## Results

### Participant Characteristics

Of the 76 participants included in the study, two were considered outliers (i.e., >3 SD above mean WMH volume) and were excluded. Thus, a total of 74 participants were included in the final analyses (see Table 1); mean (SD) age of 73.8 (2.9) years, 54 female (73%). Over half of the participants (66%) had a Fazekas score of 1, indicating low WMH load, while 36% had a score of 2 or 3, meeting the criteria for moderate to high WMH load, respectively (Fazekas et al., 1987). Mean PPA score was 0.5 (0.1), which is considered a mild risk for falling (Lord et al., 2003). Overall, participants were slightly more physically active than the average for this age group (Washburn et al., 1999), with a mean PASE score of 135.2 (68.1). Of note, 50% of participants met the criteria for mild cognitive impairment, scoring <26/30 on the MoCA (Nasreddine et al., 2005). There was a significant difference in height (*p* = 0.001) and weight (*p* = 0.007) between the analyzed sample and the parent study sample. There were no other significant differences between the two samples.

**TABLE 1:** Participant characteristics.

	**Mean (SD) or *n* (%)**			
		**Parent study**	**Participants with WMH**	
			**Total sample**	**Analyzed sample**
*N*		149	76	74
Age (years)		74.5 (3.1)	73.9 (3)	73.8 (2.9)
Female		99 (66.4%)	56 (74%)	54 (73%)
**Education**				
*High school certificate or diploma*		14 (9.4%)	9 (11.8%)	9 (12%)
*Trades or professional certificate or diploma*		24 (16.1%)	10 (13.2%)	9 (12%)
*University certificate or diploma*		13 (8.7%)	7 (9.2%)	7 (9.3%)
*University degree*		70 (47%)	37 (48.7%)	37 (49.3%)
Height (cm)		165.1 (9.4)*	151.0 (34.7)	150.7 (35.1)
Weight (kg)		74.1 (16.1)*	86 (35.9)	86 (36.4)
MMSE (/30)		28.2 (1.6)	28.4 (1.6)	28.4 (1.6)
MoCA (/30)		24.2 (3.4)	24.7 (3.5)	24.8 (3.5)
PPA		0.53 (1)	0.51 (0.9)	0.5 (1)
*Edge contrast sensitivity (dB)*		19.5 (2.3)	19.4 (2.4)	19.4 (2.5)
*Proprioception (deg)*		1.5 (1.2)	1.5 (1.1)	1.5 (1.1)
*Quadriceps strength (kg)*		29.4 (9.7)	30.0 (9.7)	30.1 (9.9)
*Hand reaction time (ms)*		241.7 (48.3)	242.3 (40.9)	241.9 (41.1)
*Postural sway (mm^2^)*		150 (112.4)	151.1 (118.8)	151.9 (120.4)
PASE		133.5 (69.5)	134.6 (67.3)	135.2 (68.1)
WMH volume (mm^3^)			4266.6 (6078.3)	3520.7 (3820.4)
Fazekas Score	1	49 (64.5%)	49 (64.5%)	49 (66.2%)
	2	19 (25%)	19 (25%)	19 (25.7%)
	3	8 (10.5%)	8 (10.5%)	6 (8.1%)

MMSE, Mini Mental State Examination; MoCA, Montreal Cognitive Assessment; PPA, Physiological Profile Assessment; PASE, Physical Activity Score for the Elderly; WMH, white matter hyperintensity. *Significant difference between parent study and participants with WMHs, p < 0.01.

### Independent Associations of WMH Volume and PASE Score With Falls Risk

**FIGURE 1:**
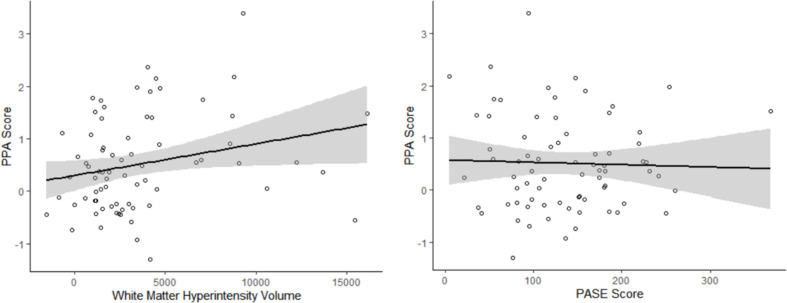
The independent associations of white matter hyperintensity (WMH) volume and Physical Activity Score for the Elderly (PASE) with Physiological Profile Assessment (PPA) score. WMH volume was associated with greater falls risk, independent of PASE score (Adjusted *R*^2^ = 5.3%). Higher PASE score was associated with lower falls risk, independent of WMH volume (Adjusted *R*^2^ = 6.9%). Age, sex, and MoCA score were included as covariates of no interest.

The independent associations of WMH volume and PASE score with PPA are illustrated in Figure 1. Greater WMH volume was significantly associated with greater falls risk (β = 0.23 ± 0.11; *p* = 0.043), independent of PA. We also found that higher PASE score was significantly associated with lower falls risk (β = −0.26 ± 0.11; *p* = 0.022), independent of WMH volume (see Table 2, model 1).

**TABLE 2:** Results of the linear regression analyses.

**Independent variables**	**Standardized β + SE**	***t-*value**	***P-*value**
**Model 1^*a*^**			0.042*
WMH Volume	0.230 (0.11)	2.063	0.043*
PASE Score	−0.256 (0.11)	–2.346	0.022*
**Model 2^*b*^**			0.012*
WMH Volume	0.381 (0.13)	2.974	0.004**
PASE Score	−0.260 (0.11)	–2.452	0.017*
WMH Volume*PASE Score	-0.268 (0.13)	–2.214	0.030*

WMH, white matter hyperintensity; PASE, Physical Activity Score for the Elderly. ^*a*^Model analyzing the independent associations of WMH volume and PASE score with PPA score. ^*b*^Model analyzing whether PASE score moderates the association between WMH volume and PPA score. *p < 0.05; **p < 0.01.

### PASE Score Moderates the Association Between WMH Volume and Falls Risk

**FIGURE 2:**
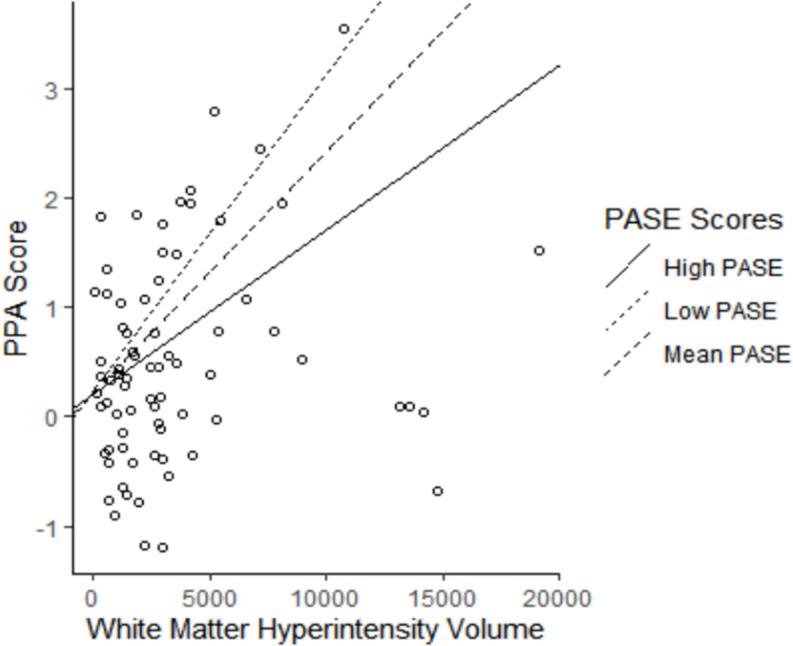
The association of white matter hyperintensity (WMH) volume with falls risk moderated by physical activity level. Higher Physical Activity Score for the Elderly (PASE) attenuated the association between WMH volume and Physiological Profile Assessment (PPA) score (Adjusted *R*^2^ = 5.8%). High PASE = 1 SD above mean; Low PASE = 1 SD below mean.

Our analyses examining if PASE score moderated the association between WMH volume and PPA score are described in Figure 2. We found that PASE score significantly moderated the association between WMH volume and falls risk (β = −0.27 ± 0.12; *p* = 0.030), whereby higher PASE score attenuated the association between WMH volume and PPA score (see Table 2, model 2). Importantly, our simple slopes analyses indicated that for participants with high PASE score, there was no longer an association between WMH and PPA score (β = 0.11 ± 0.12; *p* = 0.351).

**FIGURE 3:**
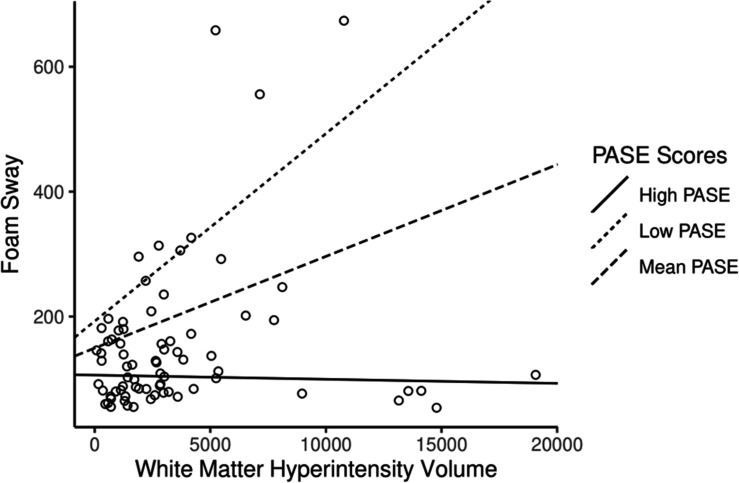
The association of white matter hyperintensity (WMH) volume with foam sway moderated by physical activity level. Higher Physical Activity Score for the Elderly (PASE) attenuated the association between WMH volume and Foam Sway (Adjusted *R*^2^ = 17.4%). High PASE = 1 SD above mean; Low PASE = 1 SD below mean.

As a result of the main findings being significant, we conducted exploratory analyses investigating whether PASE score moderated the association between WMH volume and each individual component of the PPA. We identified that PASE score significantly moderated the association between WMH volume and postural sway (β = −58.7 ± 14; *p* < 0.001), whereby higher PASE score attenuated the association between WMH and postural sway (see Table 3, model 2). The results of the simple slope analyses can be seen in Figure 3. Consistent with the PPA findings, there was no longer an association between WMH and foam sway in participants with high PASE scores (β < 0.001 ± 0.004; *p* = 0.83).

**TABLE 3:** Results of the exploratory linear regression analyses of foam sway.

**Independent variables**	**Standardized β + SE**	***t*-value**	***P*-value**
**Model 1^*a*^**			0.033*
WMH Volume	22.5 (14.0)	1.6	0.112
PASE Score	−42.3 (13.7)	–3.1	0.003**
**Model 2^*b*^**			<0.001**
WMH Volume	55.6 (14.8)	3.7	<0.001**
PASE Score	−43.2 (12.3)	–3.5	<0.001**
WMH Volume*PASE Score	−58.7 (14.0)	–4.2	<0.001**

WMH, white matter hyperintensity; PASE, Physical Activity Score for the Elderly. ^*a*^Model analyzing the independent associations of WMH volume and PASE score with foam sway. ^*b*^Model analyzing whether PASE score moderates the association between WMH volume and foam sway. *p < 0.05; **p < 0.01.

All other analyses were not significant (see Supplementary Material 2).

## Discussion

Our findings suggest PA significantly reduces the association between WMHs with falls risk after accounting for age, sex and global cognition. Thus, PA may be an important component of falls-prevention for older adults with WMHs. High volumes of WMHs substantially increase falls risk (Srikanth et al., 2009). However, our results suggest PA can reduce the association of WMHs with falls risk in older adults with low, moderate, as well as high WMH volumes. Our simple slope analysis indicates that higher PASE score attenuates the association between WMHs and falls risk; indeed, WMHs were not associated with falls risk for participants with high PASE score (>203/793). The results of our exploratory analyses identified that PA significantly moderated the association between WMH volume and the postural sway component of the PPA.

We theorize two possible underlying mechanisms by which PA may moderate the association between WMHs and falls. Disruptions to crucial neural connections as a result of WMHs has been suggested as a potential means by which WMH may lead to increased risk of falling (Zheng et al., 2011). WMHs are found to disrupt the neural connectivity of networks involved in both cognition and mobility (Murray et al., 2010; Sun et al., 2011; Ding et al., 2018), which are both associated with an increased risk of falling (Zheng et al., 2011; Nagamatsu et al., 2013). PA may counteract this deficit through neural compensation (Dao et al., 2018; Hsu et al., 2018). This pertains to the concept of less wiring more firing (Daselaar et al., 2015), whereby older adults with reduced structural connectivity are able to maintain task performance through greater activation of the remaining connections. However, this increase in neural activation requires greater resources, such as an increased oxygen supply (Lassen, 1959; Ozugur et al., 2020). PA increases blood flow, leading to greater supply of oxygen and vital nutrients to the brain (Scheinberg et al., 1954; Kleinloog et al., 2019). Thus, despite there being fewer and/or weaker neural connections in older adults with greater WMH volume, PA may support neural compensation by enabling greater activation of the remaining connections. This is highly relevant in the context of this study, as aberrant functional connectivity within and between major networks has been seen in older adults with a history of falling (Hsu et al., 2014) and is associated with poorer postural stability using the foam sway task (Crockett et al., 2017, 2019). In addition, changes in functional connectivity was significantly associated with improved mobility in older adults after 6 months of aerobic training (Hsu et al., 2017). Therefore, despite the presence of WMHs, PA may aid brain function, reducing the disruption to important networks required for falls prevention.

Physical activity has also been shown to improve muscular strength, functional mobility and balance in older adults (Langhammer et al., 2018). Reduced muscle strength, and poor functional mobility and balance are associated with greater risk of falling (Tinetti et al., 1988; Campbell et al., 1989; Wang et al., 2016). It is possible that the effect of PA on these peripheral factors may outweigh the deficit caused by WMHs, resulting in a net reduction in falls risk. This is further highlighted by our finding that, in addition to overall falls risk, PA was able to significantly moderate the association between WMHs and postural sway specifically. Therefore, it is possible that higher levels of PA in areas that improve balance, strength, and mobility may underlie the association with lower falls risk in spite of the presence of WMHs. However, PA has previously also been shown to preserve age-related decline in proprioception, which is another key risk factor for falls (Ribeiro and Oliveira, 2007). Thus, it is important to acknowledge that although the results of this study highlight the significant association between PA and postural stability, PA likely targets multiple fall risk factors simultaneously. Further research is required to greater understand the underlying mechanisms by which physical activity moderates the association between WMHs and falls risk.

### Limitations

Due to its cross-sectional design, the extent to which PA moderates the association of WMHs with falls risk cannot be generalizable to longitudinal or causal predictions of PA. Further research investigating how this relationship manifests over time would be highly beneficial. We used a self-report measure of PA. This is subject to bias and memory recall (Falck et al., 2016). Further research using an objective measure of PA is needed to support these findings. The aim of this study was to understand the role of overall PA level in moderating the relationship between WMH and falls risk. Thus, the PASE is a validated measure for this purpose (Washburn et al., 1993; Washburn and Ficker, 1999). However, using this measure, it is not possible to identify the role of each PA component on this relationship. Future research should consider identifying which, if any, components of PA may be driving this moderation. We assessed falls risk as opposed to history of falling and did not include measures of other fall risk factors, such as medication (Woolcott et al., 2009), and depression (Iaboni and Flint, 2013). While the PPA is a validated measure for assessing falls risk (Lord et al., 2003), replicating these findings with additional data of falls incidence, and other risk factors for falls, would be appropriate. Finally, the study sample was above average in PA level, and had on average a mild risk of falling. Thus, our findings may not be generalizable to older adults who are more sedentary and/or frail.

## Conclusion

Older adults with greater WMH volume are at an increased risk of falling. However, PA attenuates the association of WMHs with falls risk whereby there is no longer a statistically significant association of WMH volume with falls risk in individuals with high PA. Future work should determine the longitudinal impact of PA on WMHs and falls risk in older adults.

## Data Availability Statement

The raw data supporting the conclusions of this article will be made available by the authors, without undue reservation.

## Ethics Statement

The studies involving human participants were reviewed and approved by the Vancouver Coastal Health Research Institute, and the University of British Columbia’s Clinical Research Ethics Board (H07-00160). The patients/participants provided their written informed consent to participate in this study.

## Author Contributions

RC, CH, RT, WA, and TL-A involved in the designing and performing of the study. RC, RF, and TL-A contributed to the data analysis and were involved in the interpretation of results. RC wrote the first draft of the manuscript. RF, CH, ED, and TL-A wrote portions of the manuscript and critically reviewed the manuscript. All authors have read and approved the manuscript.

## Conflict of Interest

The authors declare that the research was conducted in the absence of any commercial or financial relationships that could be construed as a potential conflict of interest.
