# Supplementary material for: Sweat the Fall Stuff: Physical Activity Moderates the Association of White Matter Hyperintensities With Falls Risk in Older Adults
Source: Front Hum Neurosci. 2021 May 21;15:671464. doi: 10.3389/fnhum.2021.671464 (PMC8175638; doi:10.3389/fnhum.2021.671464)
Supplement: Supplementary file 1 [file Data_Sheet_1.PDF]

Supplementary Material S1 – R Version 4.0.3 Statistical Analyses Code

Ryan Stanley Falck

09/02/2021

```
#Set-up

if (!require("pacman")) install.packages("pacman")

## Loading required package: pacman

pacman::p_load(MASS,plyr,dplyr, ggplot2,psych,Hmisc,haven,tableone,PerformanceAn
alytics)

setwd("C:/Users/falckr/Desktop/Manuscripts/Ongoing Projects/PA moderates the ass
ociation of WML and Falls Risk")

data = read_sav("PASE moderates WML and PPA_no outliers.sav")

data$BMI<- data$Weight/((data$Height)^2)

data$Sex<-NA
data$Sex[data$Gender==1]<-"M"
data$Sex[data$Gender==0]<-"F"

#Participant Characteristics

vars<-dput(names(data[c(2,24,3:6,23,10,11)]))

## c("PPA_1", "Sex", "PASE", "WML_volume", "SixMWT", "Age", "BMI",
## "MOCA", "MMSE")

Table1<-CreateTableOne(vars=vars, data=data)
print(Table1,contDigits=4,missing=TRUE,quote=TRUE)

##                             ""
##  ""                          "Overall"                "Missing"
##   "n"                        "       74"              "    "
##   "PPA_1 (mean (SD))"        "   0.5115 (0.9532)"     "0.0"
##   "Sex = M (%)"              "       20 (27.0) "      "0.0"
##   "PASE (mean (SD))"         " 135.2127 (68.0806)"    "0.0"
##   "WML_volume (mean (SD))"   "3520.7027 (3820.3465)"  "0.0"
##   "SixMWT (mean (SD))"       " 492.2071 (91.3093)"    "5.4"
##   "Age (mean (SD))"          "  73.7973 (2.9469)"     "0.0"
##   "BMI (mean (SD))"          "   0.0081 (0.0152)"     "0.0"
##   "MOCA (mean (SD))"         "  24.7568 (3.4831)"     "0.0"
##   "MMSE (mean (SD))"         "  28.3919 (1.5947)"     "0.0"

#Correlations
```

```
data2<- data[c(2:4,6,10,11,23)]

cor(data2)

##                PPA_1        PASE   WML_volume         Age        MOCA
## PPA_1       1.0000000 -0.24957644  0.21955916  0.20746487 -0.06668970
## PASE       -0.2495764  1.00000000  0.13116001 -0.06242708  0.13913424
## WML_volume  0.2195592  0.13116001  1.00000000  0.15349985 -0.05759483
## Age         0.2074649 -0.06242708  0.15349985  1.00000000 -0.11297053
## MOCA       -0.0666897  0.13913424 -0.05759483 -0.11297053  1.00000000
## MMSE       -0.1623353  0.06263898 -0.10656491 -0.34724001  0.49339320
## BMI         0.2789719  0.16629097  0.03004916 -0.24796968  0.08416725
##                   MMSE         BMI
## PPA_1      -0.16233529  0.27897194
## PASE        0.06263898  0.16629097
## WML_volume -0.10656491  0.03004916
## Age        -0.34724001 -0.24796968
## MOCA        0.49339320  0.08416725
## MMSE        1.00000000  0.13711310
## BMI         0.13711310  1.00000000

chart.Correlation(data2[,1:7], histogram=FALSE, pch=19)
```

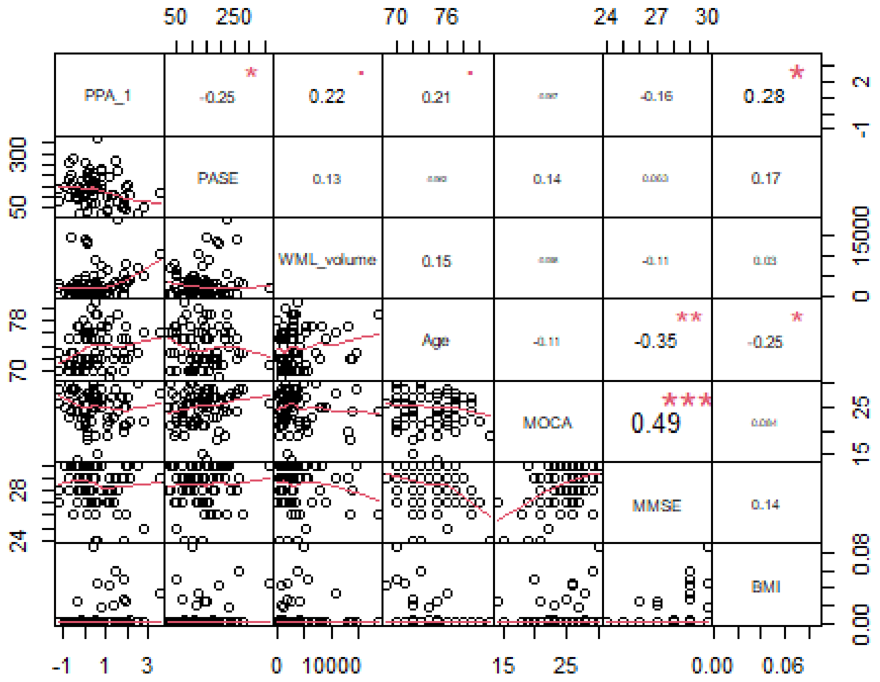


```
#Regression Analyses
```

```
#center variables
data$age.c<- scale(data$Age, center=TRUE, scale= TRUE)
data$WML.c<- scale(data$WML_volume, center=TRUE, scale= TRUE)
```

```r
data$PASE.c<- scale(data$PASE, center=TRUE, scale= TRUE)
data$MOCA.c<-scale(data$MOCA, center=TRUE, scale= TRUE)

#Primary regression model
reg1<- lm(PPA_1~PASE.c + WML.c + age.c + Sex + MOCA.c, data = data)
summary(reg1)

##
## Call:
## lm(formula = PPA_1 ~ PASE.c + WML.c + age.c + Sex + MOCA.c, data = data)
##
## Residuals:
##     Min      1Q  Median      3Q     Max
## -1.8458 -0.6706 -0.1411  0.5153  2.5321
##
## Coefficients:
##              Estimate Std. Error t value Pr(>|t|)
## (Intercept)  0.538545   0.124825   4.314 5.31e-05 ***
## PASE.c      -0.255557   0.108950  -2.346   0.0219 *
## WML.c        0.230063   0.111521   2.063   0.0429 *
## age.c        0.148572   0.108572   1.368   0.1757
## SexM        -0.100115   0.245697  -0.407   0.6849
## MOCA.c      -0.002362   0.108731  -0.022   0.9827
## ---
## Signif. codes:  0 '***' 0.001 '**' 0.01 '*' 0.05 '.' 0.1 ' ' 1
##
## Residual standard error: 0.9092 on 68 degrees of freedom
## Multiple R-squared:  0.1525, Adjusted R-squared:  0.09013
## F-statistic: 2.446 on 5 and 68 DF,  p-value: 0.04248

anova(reg1)

## Analysis of Variance Table
##
## Response: PPA_1
##           Df Sum Sq Mean Sq F value  Pr(>F)
## PASE.c     1  4.131  4.1314  4.9975 0.02867 *
## WML.c      1  4.296  4.2958  5.1963 0.02578 *
## age.c      1  1.547  1.5471  1.8714 0.17582
## Sex        1  0.137  0.1371  0.1658 0.68514
## MOCA.c     1  0.000  0.0004  0.0005 0.98274
## Residuals 68 56.215  0.8267
## ---
## Signif. codes:  0 '***' 0.001 '**' 0.01 '*' 0.05 '.' 0.1 ' ' 1

#Interaction model
reg2<-lm(PPA_1~PASE.c*WML.c + age.c + Sex + MOCA.c, data=data)
summary(reg2)

##
## Call:
## lm(formula = PPA_1 ~ PASE.c * WML.c + age.c + Sex + MOCA.c, data = data)
##
```

```
## Residuals:
##     Min      1Q  Median      3Q     Max
## -1.9783 -0.6363 -0.1016  0.6465  2.1274
##
## Coefficients:
##              Estimate Std. Error t value Pr(>|t|)
## (Intercept)   0.56419    0.12194   4.627 1.75e-05 ***
## PASE.c       -0.25986    0.10597  -2.452  0.01681 *
## WML.c         0.38080    0.12806   2.974  0.00409 **
## age.c         0.14916    0.10559   1.413  0.16239
## SexM         -0.06690    0.23941  -0.279  0.78076
## MOCA.c       -0.03256    0.10662  -0.305  0.76105
## PASE.c:WML.c -0.26756    0.12087  -2.214  0.03026 *
## ---
## Signif. codes:  0 '***' 0.001 '**' 0.01 '*' 0.05 '.' 0.1 ' ' 1
##
## Residual standard error: 0.8842 on 67 degrees of freedom
## Multiple R-squared:  0.2102, Adjusted R-squared:  0.1395
## F-statistic: 2.972 on 6 and 67 DF,  p-value: 0.01238

anova(reg2)

## Analysis of Variance Table
##
## Response: PPA_1
##              Df Sum Sq Mean Sq F value  Pr(>F)
## PASE.c        1  4.131  4.1314  5.2841 0.02465 *
## WML.c         1  4.296  4.2958  5.4943 0.02205 *
## age.c         1  1.547  1.5471  1.9787 0.16415
## Sex           1  0.137  0.1371  0.1753 0.67676
## MOCA.c        1  0.000  0.0004  0.0005 0.98225
## PASE.c:WML.c  1  3.831  3.8311  4.9000 0.03026 *
## Residuals    67 52.384  0.7819
## ---
## Signif. codes:  0 '***' 0.001 '**' 0.01 '*' 0.05 '.' 0.1 ' ' 1

#Comparison of models
anova(reg1,reg2)

## Analysis of Variance Table
##
## Model 1: PPA_1 ~ PASE.c + WML.c + age.c + Sex + MOCA.c
## Model 2: PPA_1 ~ PASE.c * WML.c + age.c + Sex + MOCA.c
##   Res.Df    RSS Df Sum of Sq   F  Pr(>F)
## 1     68 56.215
## 2     67 52.384  1    3.8311 4.9 0.03026 *
## ---
## Signif. codes:  0 '***' 0.001 '**' 0.01 '*' 0.05 '.' 0.1 ' ' 1

#Graphing the Main Effects
```

```
WML.resid<- resid(lm(WML_volume~PASE.c + age.c + Sex + MOCA.c, data = data))
PPA.resid<- resid(lm(PPA_1~PASE.c + age.c + Sex + MOCA.c, data = data))
PASE.resid<-resid(lm(PASE~WML.c + age.c + Sex + MOCA.c, data=data))
Main.effect.graph<-as.data.frame(cbind(WML.resid,PPA.resid,PASE.resid))
Main.effect.graph$WML.resid<-Main.effect.graph$WML.resid + mean(data$WML_volume)
Main.effect.graph$PPA.resid<-Main.effect.graph$PPA.resid + mean(data$PPA_1)
Main.effect.graph$PASE.resid<-Main.effect.graph$PASE.resid + mean(data$PASE)

ggplot(data = Main.effect.graph, aes(x = WML.resid, y = PPA.resid)) +
  labs(x="White Matter Hyperintensity Volume", y="PPA Score") +geom_point(shape=
1) + geom_smooth(method= 'lm',  se = TRUE, color= 'black') +
  theme_bw() + theme(panel.border = element_blank(), panel.grid.major = element_
blank(),
                     panel.grid.minor = element_blank(), axis.line = element_lin
e(colour = "black"))

## `geom_smooth()` using formula 'y ~ x'
```

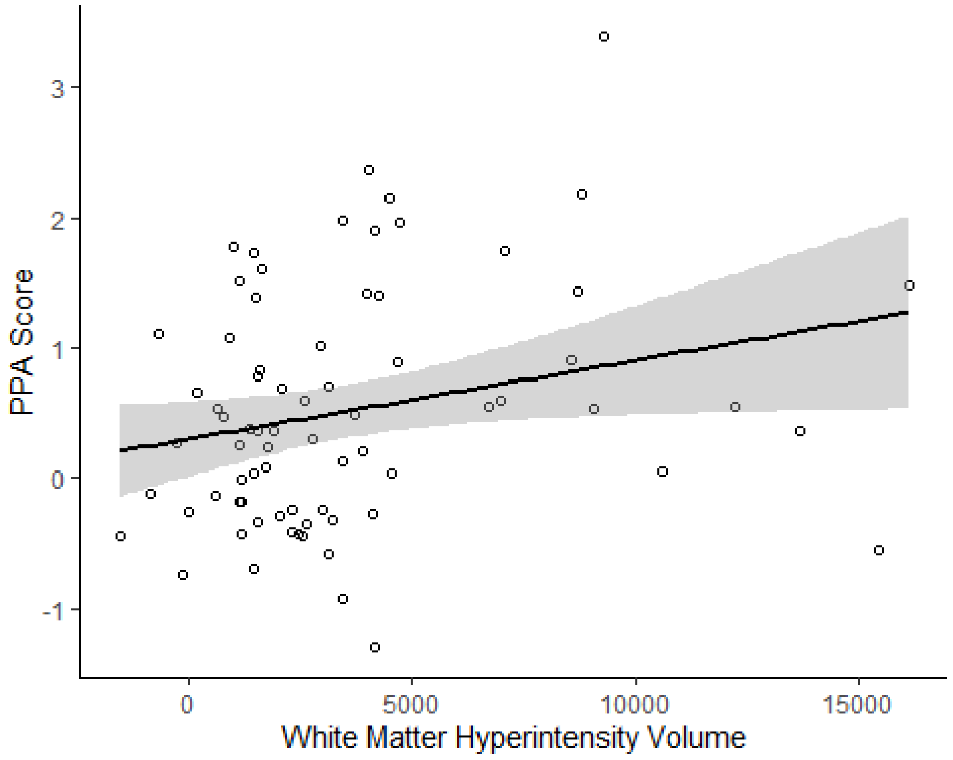


```
ggplot(data = Main.effect.graph, aes(x = PASE.resid, y = PPA.resid)) +
  labs(x="PASE Score", y="PPA Score") +geom_point(shape=1) + geom_smooth(method=
'lm',  se = TRUE, color= 'black') +
  theme_bw() + theme(panel.border = element_blank(), panel.grid.major = element_
blank(),
                     panel.grid.minor = element_blank(), axis.line = element_lin
e(colour = "black"))

## `geom_smooth()` using formula 'y ~ x'
```

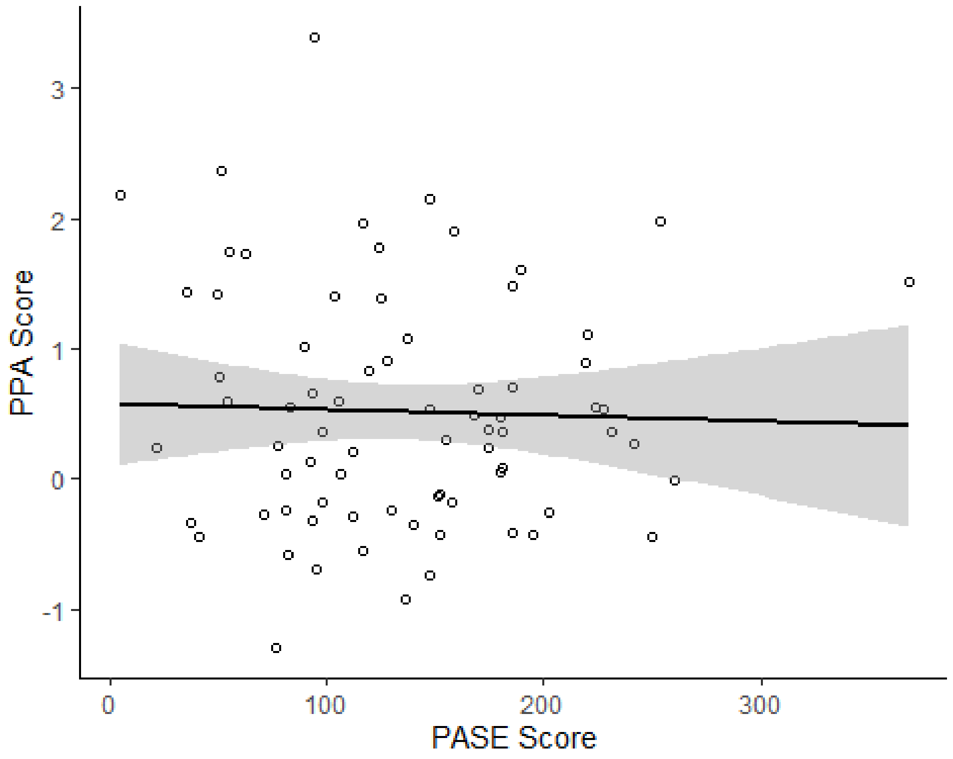


```
#Graphing the Interaction Effect

#Simple Slope Scores

data$PASE.mean<-scale(data$PASE, scale = TRUE)
data$PASE.Low<- scale(data$PASE, scale= TRUE) + 1
data$PASE.High<- scale(data$PASE, scale= TRUE) - 1
data$age.center<- scale(data$Age, center=TRUE, scale= FALSE)
data$MoCA.center<- scale(data$MOCA, center=TRUE, scale= FALSE)

reg.mean<-lm(PPA_1~PASE.mean*WML_volume + age.center + Sex + MoCA.center, data=d
ata)
reg.low<-lm(PPA_1~PASE.Low*WML_volume + age.center + Sex + MoCA.center, data=dat
a)
reg.high<-lm(PPA_1~PASE.High*WML_volume + age.center + Sex + MoCA.center, data=d
ata)
summary(reg.mean)

##
## Call:
## lm(formula = PPA_1 ~ PASE.mean * WML_volume + age.center + Sex +
##     MoCA.center, data = data)
##
## Residuals:
##     Min      1Q  Median      3Q     Max
## -1.9783 -0.6363 -0.1016  0.6465  2.1274
##
## Coefficients:
```

```
##                          Estimate Std. Error t value Pr(>|t|)
## (Intercept)           2.133e-01  1.569e-01   1.359  0.17868
## PASE.mean            -1.329e-02  1.523e-01  -0.087  0.93073
## WML_volume            9.968e-05  3.352e-05   2.974  0.00409 **
## age.center            5.061e-02  3.583e-02   1.413  0.16239
## SexM                 -6.690e-02  2.394e-01  -0.279  0.78076
## MoCA.center          -9.347e-03  3.061e-02  -0.305  0.76105
## PASE.mean:WML_volume -7.003e-05  3.164e-05  -2.214  0.03026 *
## ---
## Signif. codes:  0 '***' 0.001 '**' 0.01 '*' 0.05 '.' 0.1 ' ' 1
##
## Residual standard error: 0.8842 on 67 degrees of freedom
## Multiple R-squared:  0.2102, Adjusted R-squared:  0.1395
## F-statistic: 2.972 on 6 and 67 DF,  p-value: 0.01238
```

```r
summary(reg.low)
```

```
##
## Call:
## lm(formula = PPA_1 ~ PASE.Low * WML_volume + age.center + Sex +
##     MoCA.center, data = data)
##
## Residuals:
##     Min      1Q  Median      3Q     Max
## -1.9783 -0.6363 -0.1016  0.6465  2.1274
##
## Coefficients:
##                         Estimate Std. Error t value Pr(>|t|)
## (Intercept)          2.265e-01  2.355e-01   0.962  0.33954
## PASE.Low            -1.329e-02  1.523e-01  -0.087  0.93073
## WML_volume           1.697e-04  5.703e-05   2.976  0.00406 **
## age.center           5.061e-02  3.583e-02   1.413  0.16239
## SexM                -6.690e-02  2.394e-01  -0.279  0.78076
## MoCA.center         -9.347e-03  3.061e-02  -0.305  0.76105
## PASE.Low:WML_volume -7.003e-05  3.164e-05  -2.214  0.03026 *
## ---
## Signif. codes:  0 '***' 0.001 '**' 0.01 '*' 0.05 '.' 0.1 ' ' 1
##
## Residual standard error: 0.8842 on 67 degrees of freedom
## Multiple R-squared:  0.2102, Adjusted R-squared:  0.1395
## F-statistic: 2.972 on 6 and 67 DF,  p-value: 0.01238
```

```r
summary(reg.high)
```

```
##
## Call:
## lm(formula = PPA_1 ~ PASE.High * WML_volume + age.center + Sex +
##     MoCA.center, data = data)
##
## Residuals:
##     Min      1Q  Median      3Q     Max
## -1.9783 -0.6363 -0.1016  0.6465  2.1274
```

```
##
## Coefficients:
##                         Estimate Std. Error t value Pr(>|t|)
## (Intercept)            2.000e-01  2.005e-01   0.997   0.3221
## PASE.High             -1.329e-02  1.523e-01  -0.087   0.9307
## WML_volume             2.964e-05  3.157e-05   0.939   0.3511
## age.center             5.061e-02  3.583e-02   1.413   0.1624
## SexM                  -6.690e-02  2.394e-01  -0.279   0.7808
## MoCA.center           -9.347e-03  3.061e-02  -0.305   0.7610
## PASE.High:WML_volume -7.003e-05  3.164e-05  -2.214   0.0303 *
## ---
## Signif. codes:  0 '***' 0.001 '**' 0.01 '*' 0.05 '.' 0.1 ' ' 1
##
## Residual standard error: 0.8842 on 67 degrees of freedom
## Multiple R-squared:  0.2102, Adjusted R-squared:  0.1395
## F-statistic: 2.972 on 6 and 67 DF,  p-value: 0.01238

b0.mid <- round(reg.mean$coeff[1], digits = 3)
PASE.mid <- round(reg.mean$coeff[2], digits = 3)
WML.mid <- round(reg.mean$coeff[3], digits = 6)
interaction.mid <- round(reg.mean$coeff[6], digits = 6)

b0.low <- round(reg.low$coeff[1], digits = 3)
PASE.low <- round(reg.low$coeff[2], digits = 3)
WML.low <- round(reg.low$coeff[3], digits = 6)
interaction.low <- round(reg.low$coeff[6], digits = 6)

b0.high <- round(reg.high$coeff[1], digits = 3)
PASE.high <- round(reg.high$coeff[2], digits = 3)
WML.high <- round(reg.high$coeff[3], digits = 6)
interaction.high <- round(reg.high$coeff[6], digits = 6)

#Color Figure
ggplot(data, aes(x = WML_volume, y = PPA_1)) + geom_point(shape = 1) +
  geom_abline(aes(intercept = b0.mid, slope = WML.mid + PASE.mid*interaction.mid
, color= "Mean PASE")) +
  geom_abline(aes(intercept = b0.low, slope = WML.low + PASE.low * interaction.l
ow, color = "Low PASE")) +
  geom_abline(aes(intercept= b0.high, slope = WML.high + PASE.high*interaction.h
igh, color= "High PASE")) +
  guides(color = guide_legend((title = "PASE Scores"))) + xlab("White Matter Hyp
erintensity Volume") +
  ylab("PPA Score") + theme_bw() + theme(panel.border = element_blank(), panel.g
rid.major = element_blank(),
  panel.grid.minor = element_blank(), axis.line = element_line(colour = "black")
)
```

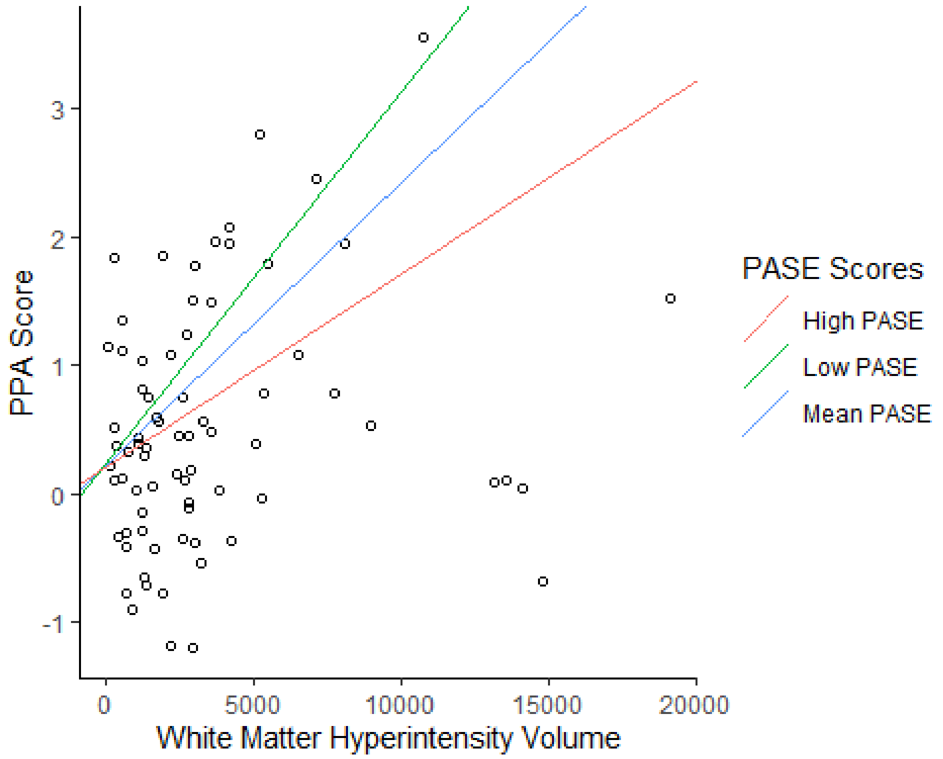


```
#Black and White
ggplot(data, aes(x = WML_volume, y = PPA_1)) + geom_point(shape = 1) +
  geom_abline(aes(intercept = b0.mid, slope = WML.mid + PASE.mid*interaction.mid
, linetype= "Mean PASE")) +
  geom_abline(aes(intercept = b0.low, slope = WML.low + PASE.low * interaction.l
ow, linetype = "Low PASE")) +
  geom_abline(aes(intercept= b0.high, slope = WML.high + PASE.high*interaction.h
igh, linetype= "High PASE")) +
  guides(linetype = guide_legend((title = "PASE Scores"))) + xlab(expression(Whi
te~Matter~Hyperintensity~Volume~(mm^3))) +
  ylab("PPA Score") + theme_bw() + theme(panel.border = element_blank(), panel.g
rid.major = element_blank(),
                                          panel.grid.minor = element_blank(), axi
s.line = element_line(colour = "black"))
```

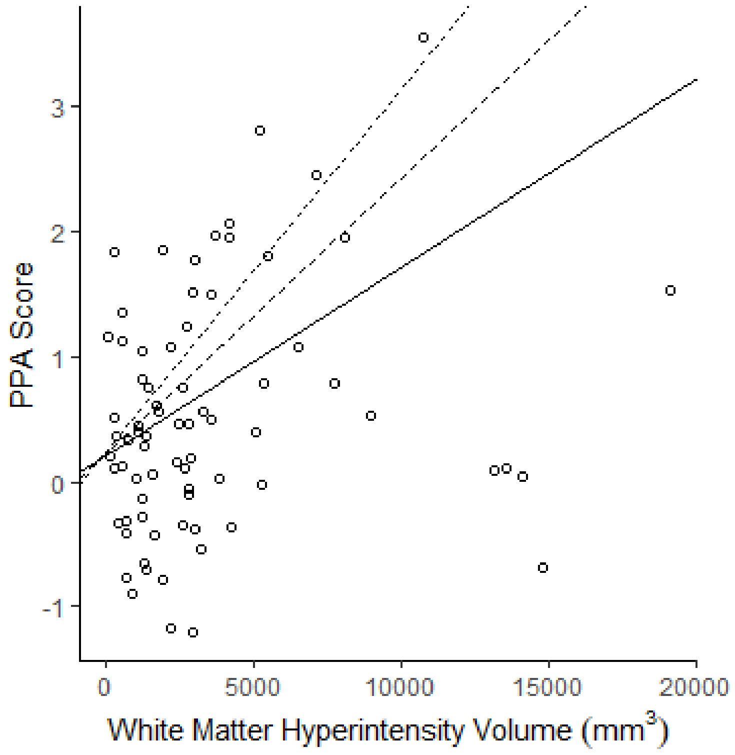
PPA Score
3
2
1
0
-1
0
5000
10000
15000
20000
White Matter Hyperintensity Volume (mm3)
PASE Scores
High PASE
Low PASE
Mean PASE
