# Supplementary material for: Sweat the Fall Stuff: Physical Activity Moderates the Association of White Matter Hyperintensities With Falls Risk in Older Adults
Source: Front Hum Neurosci. 2021 May 21;15:671464. doi: 10.3389/fnhum.2021.671464 (PMC8175638; doi:10.3389/fnhum.2021.671464)
Supplement: Supplementary file 2 [file Data_Sheet_2.PDF]

Supplementary Material S2 – R Version 4.0.3 Statistical Analyses Code

Ryan Stanley Falck

04/21/2021

```
#Participant Characteristics

vars<-dput(names(data[c(24,21,27:29,22,8,3:7)]))

## c("Age", "PASE", "Gender", "MOCA", "MMSE", "WML_volume", "PPA_1",
## "VisualContrast", "Proprioception", "Reaction_Time", "Best_Quad",
## "EO_Foam_Sway")

Table1<-CreateTableOne(vars=vars, data=data)

print(Table1,contDigits=4,missing=TRUE,quote=TRUE)

##                                  ""
##  ""                                "Overall"                "Missing"
##   "n"                              "        74"             "   "
##   "Age (mean (SD))"                "  73.7973 (2.9469)"    "0.0"
##   "PASE (mean (SD))"               " 135.2127 (68.0806)"   "0.0"
##   "Gender (mean (SD))"             "   0.2703 (0.4471)"    "0.0"
##   "MOCA (mean (SD))"               "  24.7568 (3.4831)"    "0.0"
##   "MMSE (mean (SD))"               "  28.3919 (1.5947)"    "0.0"
##   "WML_volume (mean (SD))"         "3520.7027 (3820.3465)" "0.0"
##   "PPA_1 (mean (SD))"              "   0.5115 (0.9532)"    "0.0"
##   "VisualContrast (mean (SD))"     "  19.4054 (2.4546)"    "0.0"
##   "Proprioception (mean (SD))"     "   1.4554 (1.1362)"    "0.0"
##   "Reaction_Time (mean (SD))"      " 241.8986 (41.1258)"   "0.0"
##   "Best_Quad (mean (SD))"          "  30.1096 (9.8662)"    "1.4"
##   "EO_Foam_Sway (mean (SD))"       " 151.9256 (120.3291)"  "0.0"

#Correlations
```

```
data2<- data[c(24,21,28,29,22,8,3:7)]

cor(data2, use = “pairwise.complete.obs”)
```

```
                       Age        PASE        MOCA        MMSE  WML_volume      PPA_1 VisualContrast Proprioception Reaction_Time   Best_Quad
Age             1.00000000 -0.06242708 -0.11297053 -0.34724001  0.15349985  0.2074649     0.03045357     0.06149436    0.28745511 -0.14413373
PASE           -0.06242708  1.00000000  0.13913424  0.06263898  0.13116001 -0.2495764    -0.14002414    -0.06193346   -0.05753464  0.19418898
MOCA           -0.11297053  0.13913424  1.00000000  0.49339320 -0.05759483 -0.0666897     0.04373703    -0.06923734   -0.04418394  0.12245044
MMSE           -0.34724001  0.06263898  0.49339320  1.00000000 -0.10656491 -0.1623353     0.02884840    -0.07036296   -0.19518873  0.05168954
WML_volume      0.15349985  0.13116001 -0.05759483 -0.10656491  1.00000000  0.2195592    -0.26982372    -0.07443901    0.20070450  0.17354743
PPA_1           0.20746487 -0.24957644 -0.06668970 -0.16233529  0.21955916  1.0000000    -0.25183954     0.25994549    0.45264029 -0.22377519
VisualContrast  0.03045357 -0.14002414  0.04373703  0.02884840 -0.26982372 -0.2518395     1.00000000     0.09154298   -0.03021472 -0.25937911
Proprioception  0.06149436 -0.06193346 -0.06923734 -0.07036296 -0.07443901  0.2599455     0.09154298     1.00000000    0.01675845 -0.16535373
Reaction_Time   0.28745511 -0.05753464 -0.04418394 -0.19518873  0.20070450  0.4526403    -0.03021472     0.01675845    1.00000000 -0.35006505
Best_Quad      -0.14413373  0.19418898  0.12245044  0.05168954  0.17354743 -0.2237752    -0.25937911    -0.16535373   -0.35006505  1.00000000
EO_Foam_Sway    0.08371410 -0.30586858  0.04018341 -0.05095433  0.16632827  0.7764084    -0.01746791     0.09085619    0.02407818 -0.02445783
               EO_Foam_Sway
Age              0.08371410
PASE            -0.30586858
MOCA             0.04018341
MMSE            -0.05095433
WML_volume       0.16632827
PPA_1            0.77640844
VisualContrast  -0.01746791
Proprioception   0.09085619
Reaction_Time    0.02407818
Best_Quad       -0.02445783
EO_Foam_Sway     1.00000000
```

```
chart.Correlation(data2[,1:7], histogram=FALSE, pch=19)
```

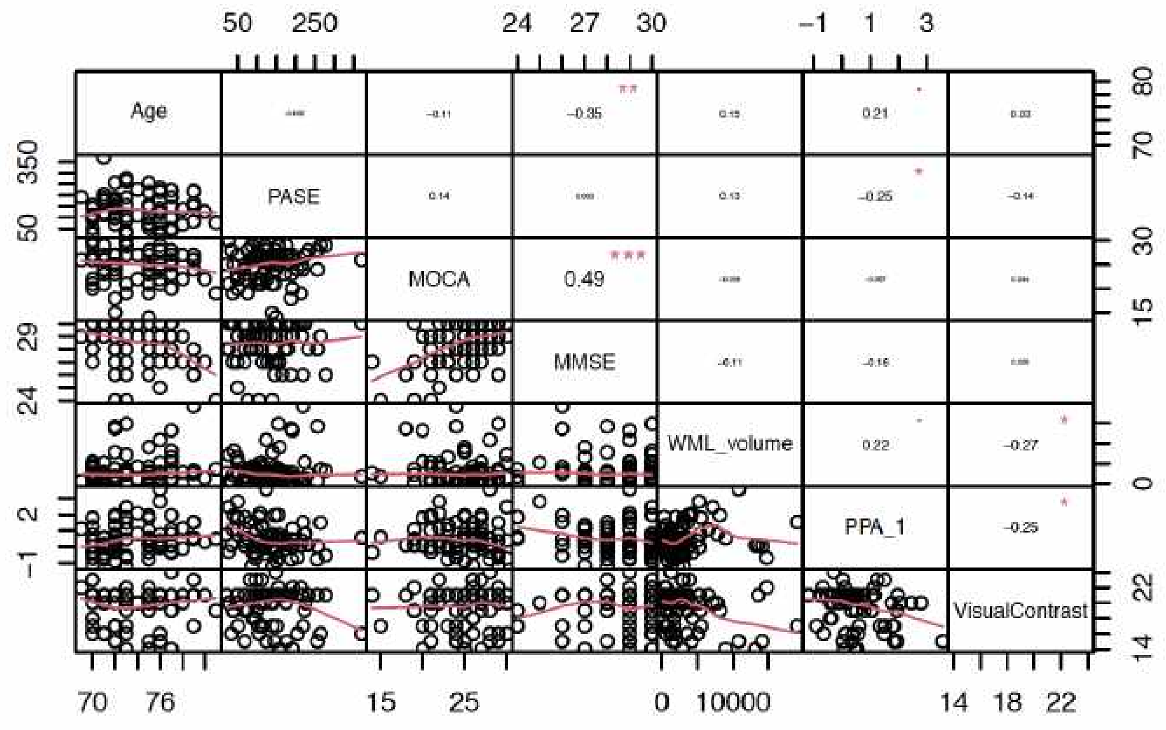


```
#Regression Analyses
```

```r
#center variables
data$age.c<- scale(data$Age, center=TRUE, scale= TRUE)
data$WML.c<- scale(data$WML_volume, center=TRUE, scale= TRUE)
data$PASE.c<- scale(data$PASE, center=TRUE, scale= TRUE)
data$MOCA.c<-scale(data$MOCA, center=TRUE, scale= TRUE)

#Regression model:Edge Contrast Sensitivity
reg1<- lm(VisualContrast~PASE.c + WML.c + age.c + Sex + MOCA.c, data =
data)
summary(reg1)
```

```
##Call:
## lm(formula = VisualContrast ~ PASE.c + WML.c + age.c + Sex +
##     MOCA.c, data = data)
##
## Residuals:
##     Min      1Q  Median      3Q     Max
## -5.4813 -1.6906  0.8169  1.4420  4.2150
##
## Coefficients:
##             Estimate Std. Error t value Pr(>|t|)
## (Intercept)  19.5559     0.3312  59.048   <2e-16 ***
## PASE.c       -0.2532     0.2891  -0.876    0.384
## WML.c        -0.5932     0.2959  -2.005    0.049 *
## age.c         0.1748     0.2881   0.607    0.546
## SexM         -0.5567     0.6519  -0.854    0.396
## MOCA.c        0.1038     0.2885   0.360    0.720
## ---
## Signif. codes:  0 '***' 0.001 '**' 0.01 '*' 0.05 '.' 0.1 ' ' 1

## Residual standard error: 2.412 on 68 degrees of freedom
## Multiple R-squared:  0.1003,    Adjusted R-squared:  0.03411
## F-statistic: 1.516 on 5 and 68 DF,  p-value: 0.1965
anova(reg1)
```

```
## Analysis of Variance Table

## Response: VisualContrast
##            Df Sum Sq Mean Sq F value  Pr(>F)
## PASE.c      1   8.62  8.6238  1.4818 0.22770
## WML.c       1  28.30 28.2983  4.8625 0.03083 *
## age.c       1   1.81  1.8053  0.3102 0.57938
## Sex         1   4.62  4.6199  0.7938 0.37608
## MOCA.c      1   0.75  0.7533  0.1294 0.72013
## Residuals 68 395.74  5.8197
## ---
## Signif. codes:  0 '***' 0.001 '**' 0.01 '*' 0.05 '.' 0.1 ' ' 1
#Interaction model
reg2<-lm(VisualContrast ~PASE.c*WML.c + age.c + Sex + MOCA.c, data=data)
summary(reg2)
## Call:
## lm(formula = VisualContrast ~ PASE.c * WML.c + age.c + Sex +
##     MOCA.c, data = data)

## Residuals:
##     Min      1Q  Median      3Q     Max
## -5.4977 -1.1297  0.8515  1.4028  4.0912
##
## Coefficients:
##               Estimate Std. Error t value Pr(>|t|)
## (Intercept)  19.58506    0.33306  58.804   <2e-16 ***
## PASE.c       -0.25814    0.28943  -0.892    0.376
## WML.c        -0.42165    0.34976  -1.206    0.232
## age.c         0.17547    0.28838   0.608    0.545
## SexM         -0.51894    0.65389  -0.794    0.430
```

```
## MOCA.c        0.06943    0.29120   0.238    0.812
## PASE.c:WML.c -0.30453    0.33012  -0.922    0.360
## ---
## Signif. codes:  0 '***' 0.001 '**' 0.01 '*' 0.05 '.' 0.1 ' ' 1

## Residual standard error: 2.415 on 67 degrees of freedom
## Multiple R-squared:  0.1115,     Adjusted R-squared:  0.03199
## F-statistic: 1.402 on 6 and 67 DF,  p-value: 0.2269
anova(reg2)
## Analysis of Variance Table
##
## Response: VisualContrast
##              Df Sum Sq Mean Sq F value  Pr(>F)
## PASE.c        1   8.62  8.6238  1.4786 0.22826
## WML.c         1  28.30 28.2983  4.8519 0.03106 *
## age.c         1   1.81  1.8053  0.3095 0.57982
## Sex           1   4.62  4.6199  0.7921 0.37665
## MOCA.c        1   0.75  0.7533  0.1292 0.72044
## PASE.c:WML.c  1   4.96  4.9632  0.8510 0.35959
## Residuals    67 390.77  5.8324
## ---
## Signif. codes:  0 '***' 0.001 '**' 0.01 '*' 0.05 '.' 0.1 ' ' 1
#Comparison of models
anova(reg1,reg2)
## Analysis of Variance Table
##
## Model 1: VisualContrast ~ PASE.c + WML.c + age.c + Sex + MOCA.c
## Model 2: VisualContrast ~ PASE.c * WML.c + age.c + Sex + MOCA.c
##   Res.Df    RSS Df Sum of Sq      F Pr(>F)
## 1     68 395.74
```

```
## 2     67 390.77  1    4.9632 0.851 0.3596
```

```r
#Regression model:Quad Strength
reg1<- lm(Best_Quad~PASE.c + WML.c + age.c + Sex + MOCA.c, data = data)
summary(reg1)

## Call:

## lm(formula = Best_Quad ~ PASE.c + WML.c + age.c + Sex + MOCA.c,

##     data = data)

##

## Residuals:

##     Min      1Q  Median      3Q     Max

## -22.810  -4.354   1.178   4.258  23.066

##

## Coefficients:

##             Estimate Std. Error t value Pr(>|t|)

## (Intercept)  26.6923     1.0656  25.049  < 2e-16 ***

## PASE.c        1.1725     0.9299   1.261   0.2117

## WML.c         0.4414     0.9556   0.462   0.6457

## age.c        -1.7691     0.9354  -1.891   0.0629 .

## SexM         13.3634     2.1553   6.200 3.97e-08 ***

## MOCA.c        1.6752     0.9383   1.785   0.0787 .

## ---

## Signif. codes:  0 '***' 0.001 '**' 0.01 '*' 0.05 '.' 0.1 ' ' 1

##

## Residual standard error: 7.76 on 67 degrees of freedom

##   (1 observation deleted due to missingness)

## Multiple R-squared:  0.4243,     Adjusted R-squared:  0.3814

## F-statistic: 9.877 on 5 and 67 DF,  p-value: 4.174e-07

anova(reg1)

## Analysis of Variance Table


## Response: Best_Quad
```

```
##           Df Sum Sq Mean Sq F value    Pr(>F)
## PASE.c     1  264.3  264.29  4.3888   0.03996 *
## WML.c      1  155.4  155.37  2.5800   0.11292
## age.c      1  175.3  175.34  2.9117   0.09257 .
## Sex        1 2187.0 2187.03 36.3181 7.979e-08 ***
## MOCA.c     1  191.9  191.93  3.1872   0.07874 .
## Residuals 67 4034.7   60.22
## ---
## Signif. codes:  0 ‘***’ 0.001 ‘**’ 0.01 ‘*’ 0.05 ‘.’ 0.1 ‘ ’ 1
#Interaction model
reg2<-lm(Best_Quad ~PASE.c*WML.c + age.c + Sex + MOCA.c, data=data)
summary(reg2)
## Call:
## lm(formula = Best_Quad ~ PASE.c * WML.c + age.c + Sex + MOCA.c,
##     data = data)
##
## Residuals:
##      Min       1Q   Median       3Q      Max
## -21.6507  -4.5908   0.7064   4.4097  23.2177
##
## Coefficients:
##               Estimate Std. Error t value Pr(>|t|)
## (Intercept)  26.60734    1.07295  24.798  < 2e-16 ***
## PASE.c        1.18679    0.93218   1.273   0.2074
## WML.c        -0.05751    1.13014  -0.051   0.9596
## age.c        -1.77205    0.93752  -1.890   0.0631 .
## SexM         13.25753    2.16408   6.126  5.6e-08 ***
## MOCA.c        1.77603    0.94827   1.873   0.0655 .
## PASE.c:WML.c  0.88434    1.06325   0.832   0.4086
## ---
```

```
## Signif. codes:  0 '***' 0.001 '**' 0.01 '*' 0.05 '.' 0.1 ' ' 1
##
## Residual standard error: 7.778 on 66 degrees of freedom
##   (1 observation deleted due to missingness)
## Multiple R-squared:  0.4303,     Adjusted R-squared:  0.3785
## F-statistic: 8.308 on 6 and 66 DF,  p-value: 1.028e-06
anova(reg2)
## Analysis of Variance Table
##
## Response: Best_Quad
##               Df Sum Sq Mean Sq F value    Pr(>F)
## PASE.c         1  264.3  264.29  4.3686   0.04046 *
## WML.c          1  155.4  155.37  2.5682   0.11381
## age.c          1  175.3  175.34  2.8983   0.09338 .
## Sex            1 2187.0 2187.03 36.1510 8.811e-08 ***
## MOCA.c         1  191.9  191.93  3.1726   0.07949 .
## PASE.c:WML.c  1   41.8   41.85  0.6918   0.40856
## Residuals     66 3992.8   60.50
## ---
## Signif. codes:  0 '***' 0.001 '**' 0.01 '*' 0.05 '.' 0.1 ' ' 1
#Comparison of models
anova(reg1,reg2)
## Analysis of Variance Table
##
## Model 1: Best_Quad ~ PASE.c + WML.c + age.c + Sex + MOCA.c
## Model 2: Best_Quad ~ PASE.c * WML.c + age.c + Sex + MOCA.c
##   Res.Df    RSS Df Sum of Sq      F Pr(>F)
## 1     67 4034.7
## 2     66 3992.8  1     41.85 0.6918 0.4086
```

```
#Regression model:Proprioception
reg1<- lm(Proprioception~PASE.c + WML.c + age.c + Sex + MOCA.c, data = data)
summary(reg1)

## Call:

## lm(formula = Proprioception ~ PASE.c + WML.c + age.c + Sex +

##     MOCA.c, data = data)

##

## Residuals:

##     Min      1Q  Median      3Q     Max

## -1.5722 -0.7531 -0.2818  0.4747  3.8001

##

## Coefficients:

##             Estimate Std. Error t value Pr(>|t|)

## (Intercept)  1.53364    0.15926   9.630  2.5e-14 ***

## PASE.c      -0.03720    0.13901  -0.268    0.790

## WML.c       -0.06646    0.14229  -0.467    0.642

## age.c        0.07535    0.13853   0.544    0.588

## SexM        -0.28945    0.31348  -0.923    0.359

## MOCA.c      -0.08149    0.13873  -0.587    0.559

## ---

## Signif. codes:  0 '***' 0.001 '**' 0.01 '*' 0.05 '.' 0.1 ' ' 1

##

## Residual standard error: 1.16 on 68 degrees of freedom

## Multiple R-squared:  0.02896,    Adjusted R-squared:  -0.04244

## F-statistic: 0.4057 on 5 and 68 DF,  p-value: 0.8433

anova(reg1)

## Analysis of Variance Table

##

## Response: Proprioception

##           Df Sum Sq Mean Sq F value Pr(>F)
```

```
## PASE.c     1  0.361 0.36149  0.2686 0.6059
## WML.c      1  0.422 0.42171  0.3134 0.5775
## age.c      1  0.457 0.45663  0.3393 0.5622
## Sex        1  1.025 1.02549  0.7620 0.3858
## MOCA.c     1  0.464 0.46431  0.3450 0.5589
## Residuals 68 91.513 1.34578
#Interaction model
reg2<-lm(Proprioception ~PASE.c*WML.c + age.c + Sex + MOCA.c, data=data)
summary(reg2)
## Call:
## lm(formula = Proprioception ~ PASE.c * WML.c + age.c + Sex +
##     MOCA.c, data = data)
##
## Residuals:
##     Min      1Q  Median      3Q     Max
## -1.5780 -0.7517 -0.2752  0.5063  3.7950
##
## Coefficients:
##              Estimate Std. Error t value Pr(>|t|)
## (Intercept)   1.53127    0.16115   9.502 4.86e-14 ***
## PASE.c       -0.03680    0.14004  -0.263    0.794
## WML.c        -0.08036    0.16923  -0.475    0.636
## age.c         0.07529    0.13953   0.540    0.591
## SexM         -0.29251    0.31638  -0.925    0.359
## MOCA.c       -0.07870    0.14089  -0.559    0.578
## PASE.c:WML.c  0.02466    0.15973   0.154    0.878
## ---
## Signif. codes:  0 ‘***’ 0.001 ‘**’ 0.01 ‘*’ 0.05 ‘.’ 0.1 ‘ ’ 1
##
## Residual standard error: 1.168 on 67 degrees of freedom
```

```
## Multiple R-squared:  0.02931,    Adjusted R-squared:  -0.05762

## F-statistic: 0.3372 on 6 and 67 DF,  p-value: 0.9149

anova(reg2)

## Analysis of Variance Table

##

## Response: Proprioception

##                Df Sum Sq Mean Sq F value Pr(>F)

## PASE.c          1  0.361 0.36149  0.2648 0.6086

## WML.c           1  0.422 0.42171  0.3089 0.5802

## age.c           1  0.457 0.45663  0.3344 0.5650

## Sex             1  1.025 1.02549  0.7511 0.3892

## MOCA.c          1  0.464 0.46431  0.3401 0.5618

## PASE.c:WML.c  1  0.033 0.03255  0.0238 0.8778

## Residuals    67 91.481 1.36538

#Comparison of models
anova(reg1,reg2)

## Analysis of Variance Table

##

## Model 1: Proprioception ~ PASE.c + WML.c + age.c + Sex + MOCA.c

## Model 2: Proprioception ~ PASE.c * WML.c + age.c + Sex + MOCA.c

##   Res.Df    RSS Df Sum of Sq      F Pr(>F)

## 1     68 91.513

## 2     67 91.481  1   0.03255 0.0238 0.8778
```

```r
#Regression model:Hand Reaction Time
reg1<- lm(Reaction_Time~PASE.c + WML.c + age.c + Sex + MOCA.c, data = data)
summary(reg1)

## Call:

## lm(formula = Reaction_Time ~ PASE.c + WML.c + age.c + Sex + MOCA.c,

##     data = data)

##

## Residuals:

##     Min      1Q  Median      3Q     Max

## -65.747 -29.203  -4.633  24.020 117.585

##

## Coefficients:

##             Estimate Std. Error t value Pr(>|t|)

## (Intercept) 245.6684     5.4461  45.109   <2e-16 ***

## PASE.c       -2.3265     4.7535  -0.489   0.6261

## WML.c         8.3172     4.8656   1.709   0.0919 .

## age.c        10.6803     4.7370   2.255   0.0274 *

## SexM        -13.9479    10.7198  -1.301   0.1976

## MOCA.c       -0.4187     4.7439  -0.088   0.9299

## ---

## Signif. codes:  0 ‘***’ 0.001 ‘**’ 0.01 ‘*’ 0.05 ‘.’ 0.1 ‘ ’ 1

##

## Residual standard error: 39.67 on 68 degrees of freedom

## Multiple R-squared:  0.1333,     Adjusted R-squared:  0.06956

## F-statistic: 2.092 on 5 and 68 DF,  p-value: 0.07702

anova(reg1)

## Analysis of Variance Table

##

## Response: Reaction_Time

##           Df Sum Sq Mean Sq F value  Pr(>F)
```

```
## PASE.c      1    409   408.7  0.2597 0.61197
## WML.c       1   5448  5448.3  3.4621 0.06711 .
## age.c       1   7934  7934.2  5.0418 0.02800 *
## Sex         1   2654  2653.5  1.6862 0.19849
## MOCA.c      1     12    12.3  0.0078 0.92994
## Residuals 68 107011  1573.7
## ---
## Signif. codes:  0 '***' 0.001 '**' 0.01 '*' 0.05 '.' 0.1 ' ' 1
```

```
#Interaction model
reg2<-lm(Reaction_Time ~PASE.c*WML.c + age.c + Sex + MOCA.c, data=data)
summary(reg2)
```

```
## Call:
## lm(formula = Reaction_Time ~ PASE.c * WML.c + age.c + Sex + MOCA.c,
##     data = data)
##
## Residuals:
##    Min     1Q Median     3Q    Max
## -70.85 -31.11  -2.04  24.58 116.44
##
## Coefficients:
##               Estimate Std. Error t value Pr(>|t|)
## (Intercept)   245.1194     5.4661  44.844   <2e-16 ***
## PASE.c         -2.2343     4.7502  -0.470   0.6396
## WML.c           5.0899     5.7403   0.887   0.3784
## age.c          10.6678     4.7329   2.254   0.0275 *
## SexM          -14.6590    10.7316  -1.366   0.1765
## MOCA.c          0.2278     4.7791   0.048   0.9621
## PASE.c:WML.c    5.7283     5.4179   1.057   0.2942
## ---
## Signif. codes:  0 '***' 0.001 '**' 0.01 '*' 0.05 '.' 0.1 ' ' 1
```

```
## Residual standard error: 39.64 on 67 degrees of freedom

## Multiple R-squared:  0.1475,     Adjusted R-squared:  0.07117

## F-statistic: 1.932 on 6 and 67 DF,  p-value: 0.08823

anova(reg2)

## Analysis of Variance Table

##

## Response: Reaction_Time

##               Df Sum Sq Mean Sq F value  Pr(>F)

## PASE.c         1    409   408.7  0.2602 0.61168

## WML.c          1   5448  5448.3  3.4681 0.06695 .

## age.c          1   7934  7934.2  5.0505 0.02792 *

## Sex            1   2654  2653.5  1.6891 0.19817

## MOCA.c         1     12    12.3  0.0078 0.92988

## PASE.c:WML.c   1   1756  1756.1  1.1179 0.29418

## Residuals     67 105254  1571.0

## ---

## Signif. codes:  0 '***' 0.001 '**' 0.01 '*' 0.05 '.' 0.1 ' ' 1

#Comparison of models
anova(reg1,reg2)

## Analysis of Variance Table

##

## Model 1: Reaction_Time ~ PASE.c + WML.c + age.c + Sex + MOCA.c

## Model 2: Reaction_Time ~ PASE.c * WML.c + age.c + Sex + MOCA.c

##   Res.Df    RSS Df Sum of Sq      F Pr(>F)

## 1     68 107011

## 2     67 105254  1    1756.1 1.1179 0.2942
```

```
#Regression model:Foam Sway
reg1<- lm(EO_Foam_Sway~PASE.c + WML.c + age.c + Sex + MOCA.c, data = data)
summary(reg1)

## Call:

## lm(formula = EO_Foam_Sway ~ PASE.c + WML.c + age.c + Sex + MOCA.c,

##     data = data)

##

## Residuals:

##     Min      1Q  Median      3Q     Max

## -159.49  -63.39  -17.54   38.57  433.12

##

## Coefficients:

##             Estimate Std. Error t value Pr(>|t|)

## (Intercept)  143.742     15.683   9.165  1.7e-13 ***

## PASE.c       -42.271     13.688  -3.088  0.00292 **

## WML.c         22.530     14.011   1.608  0.11247

## age.c          4.833     13.641   0.354  0.72419

## SexM          30.280     30.869   0.981  0.33012

## MOCA.c        13.886     13.661   1.016  0.31300

## ---

## Signif. codes:  0 ‘***’ 0.001 ‘**’ 0.01 ‘*’ 0.05 ‘.’ 0.1 ‘ ’ 1

##

## Residual standard error: 114.2 on 68 degrees of freedom

## Multiple R-squared:  0.1604,    Adjusted R-squared:  0.09872

## F-statistic: 2.599 on 5 and 68 DF,  p-value: 0.03281

anova(reg1)

## Analysis of Variance Table

##

## Response: EO_Foam_Sway

##           Df Sum Sq Mean Sq F value   Pr(>F)
```

```
## PASE.c     1  98886   98886  7.5776 0.007571 **
## WML.c      1  45837   45837  3.5125 0.065206 .
## age.c      1   1025    1025  0.0785 0.780176
## Sex        1  10360   10360  0.7939 0.376061
## MOCA.c     1  13484   13484  1.0333 0.312998
## Residuals 68 887383   13050
## ---
## Signif. codes:  0 '***' 0.001 '**' 0.01 '*' 0.05 '.' 0.1 ' ' 1
```

```
#Interaction model
reg2<-lm(EO_Foam_Sway ~PASE.c*WML.c + age.c + Sex + MOCA.c, data=data)
summary(reg2)
```

```
## Call:
## lm(formula = EO_Foam_Sway ~ PASE.c * WML.c + age.c + Sex + MOCA.c,
##     data = data)
##
## Residuals:
##     Min      1Q  Median      3Q     Max
## -203.26  -58.69  -18.00   43.53  361.76
##
## Coefficients:
##               Estimate Std. Error t value Pr(>|t|)
## (Intercept)   149.364     14.129  10.572 6.43e-16 ***
## PASE.c        -43.214     12.278  -3.520 0.000782 ***
## WML.c          55.579     14.837   3.746 0.000376 ***
## age.c           4.961     12.234   0.406 0.686360
## SexM           37.562     27.739   1.354 0.180253
## MOCA.c          7.266     12.353   0.588 0.558362
## PASE.c:WML.c  -58.661     14.004  -4.189 8.38e-05 ***
## ---
## Signif. codes:  0 '***' 0.001 '**' 0.01 '*' 0.05 '.' 0.1 ' ' 1
```

```
##
## Residual standard error: 102.4 on 67 degrees of freedom
## Multiple R-squared:  0.3347,     Adjusted R-squared:  0.2751
## F-statistic: 5.617 on 6 and 67 DF,  p-value: 9.071e-05
anova(reg2)
## Analysis of Variance Table
##
## Response: EO_Foam_Sway
##               Df Sum Sq Mean Sq F value    Pr(>F)
## PASE.c         1  98886   98886  9.4214 0.003095 **
## WML.c          1  45837   45837  4.3671 0.040438 *
## age.c          1   1025    1025  0.0976 0.755682
## Sex            1  10360   10360  0.9871 0.324029
## MOCA.c         1  13484   13484  1.2847 0.261072
## PASE.c:WML.c   1 184158  184158 17.5457 8.38e-05 ***
## Residuals     67 703225   10496
## ---
## Signif. codes:  0 '***' 0.001 '**' 0.01 '*' 0.05 '.' 0.1 ' ' 1
#Comparison of models
anova(reg1,reg2)
## Analysis of Variance Table

## Model 1: EO_Foam_Sway ~ PASE.c + WML.c + age.c + Sex + MOCA.c
## Model 2: EO_Foam_Sway ~ PASE.c * WML.c + age.c + Sex + MOCA.c
##   Res.Df    RSS Df Sum of Sq      F   Pr(>F)
## 1     68 887383
## 2     67 703225  1    184158 17.546 8.38e-05 ***
## ---
## Signif. codes:  0 '***' 0.001 '**' 0.01 '*' 0.05 '.' 0.1 ' ' 1
```

```r
#Graphing the Main Effects

WML.resid<- resid(lm(WML_volume~PASE.c + age.c + Sex + MOCA.c, data = data))
Sway.resid<- resid(lm(EO_Foam_Sway ~PASE.c + age.c + Sex + MOCA.c, data = data))
PASE.resid<-resid(lm(PASE~WML.c + age.c + Sex + MOCA.c, data=data))
Main.effect.graph<-as.data.frame(cbind(WML.resid,Sway.resid,PASE.resid))
Main.effect.graph$WML.resid<-Main.effect.graph$WML.resid + mean(data$WML_volume)
Main.effect.graph$Sway.resid<-Main.effect.graph$Sway.resid + mean(data$ EO_Foam_
Sway)
Main.effect.graph$PASE.resid<-Main.effect.graph$PASE.resid + mean(data$PASE)

ggplot(data = Main.effect.graph, aes(x = WML.resid, y = Sway.resid)) +
  labs(x="White Matter Hyperintensity Volume", y="Foam Sway (mm²)") +geom_point(s
hape=1) + geom_smooth(method= 'lm',  se = TRUE, color= 'black') +
  theme_bw() + theme(panel.border = element_blank(), panel.grid.major = element_
blank(),
                          panel.grid.minor = element_blank(), axis.line = element_lin
e(colour = "black"))

## `geom_smooth()` using formula 'y ~ x'
```

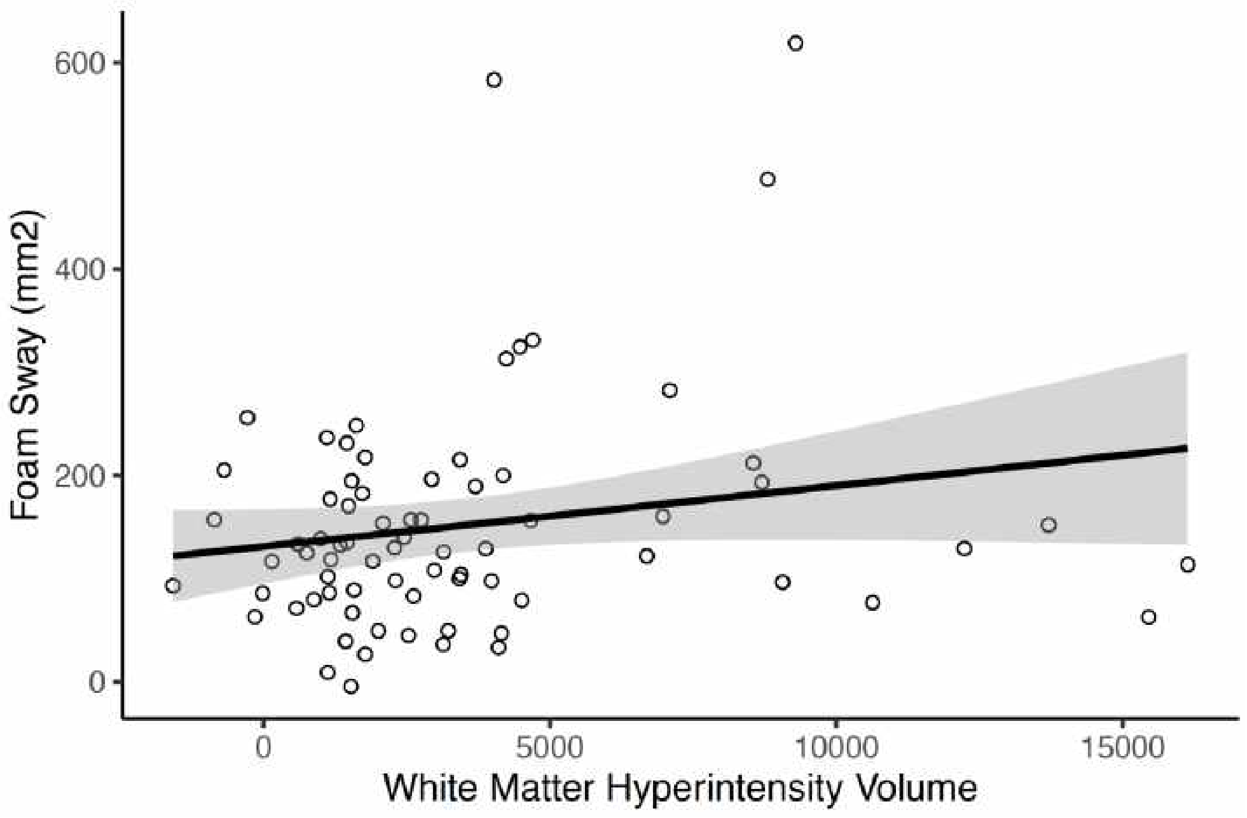


```r
ggplot(data = Main.effect.graph, aes(x = PASE.resid, y = Sway.resid)) +
  labs(x="PASE Score", y=" Foam Sway (mm²)") +geom_point(shape=1) + geom_smooth(m
ethod= 'lm',  se = TRUE, color= 'black') +
  theme_bw() + theme(panel.border = element_blank(), panel.grid.major = element_
blank(),
                          panel.grid.minor = element_blank(), axis.line = element_lin
e(colour = "black"))
```

```
## `geom_smooth()` using formula 'y ~ x'
```

`geom_smooth()` using formula 'y ~ x'

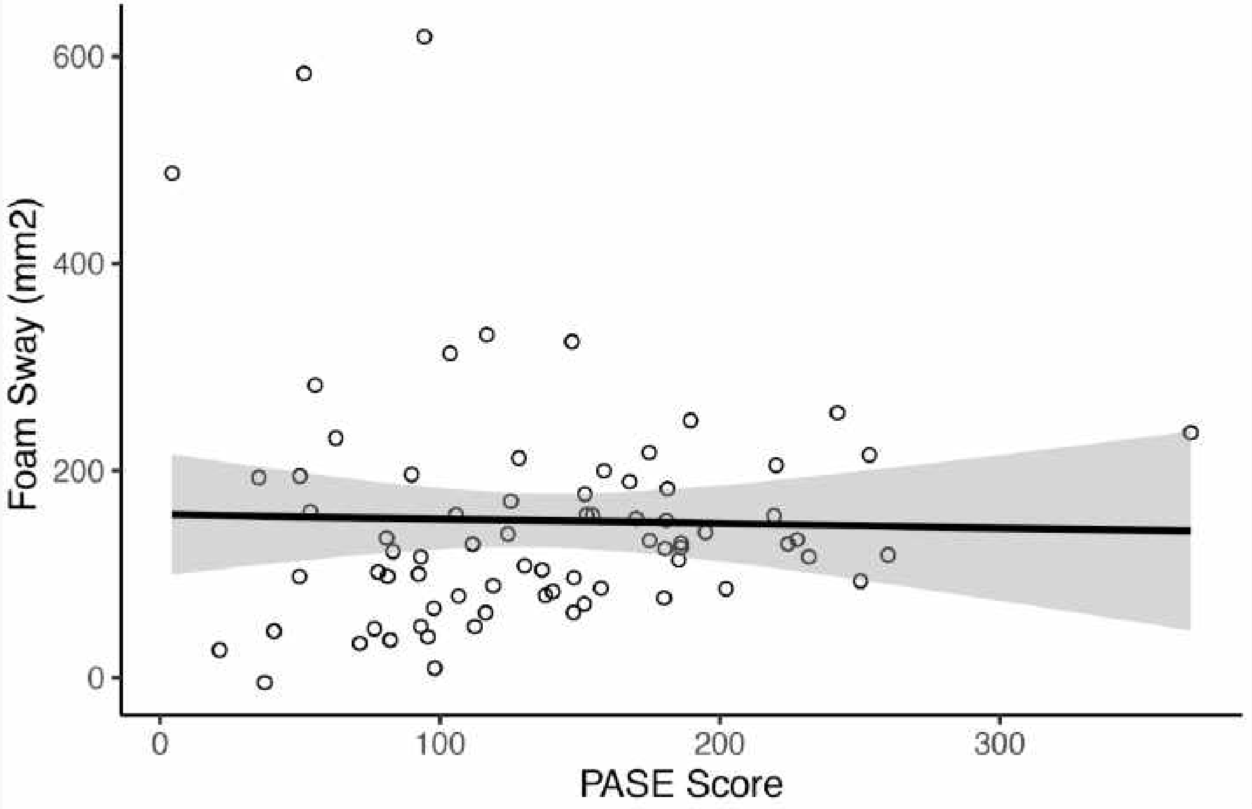


```
#Graphing the Interaction Effect

#Simple Slope Scores

data$PASE.mean.c<-scale(data$PASE, center=TRUE, scale=FALSE)
data$PASE.Low<- data$PASE.mean.c + sd(data$PASE)
data$PASE.High<- data$PASE.mean.c - sd(data$PASE)
data$age.center<- scale(data$Age, center=TRUE, scale=FALSE)
data$MoCA.center<- scale(data$MOCA, center=TRUE, scale=FALSE)
data$WML_volume.c<-scale(data$WML_volume, center=TRUE, scale=FALSE)

reg.mean<-lm(EO_Foam_Sway~PASE.mean.c*WML_volume.c + age.center + Sex +
MoCA.center, data=data)
reg.low<-lm(EO_Foam_Sway~PASE.Low*WML_volume.c + age.center + Sex + MoCA.center,
data=data)
reg.high<-lm(EO_Foam_Sway~PASE.High*WML_volume.c + age.center + Sex +
MoCA.center, data=data)
```

summary(reg.mean)

```
## Call:
## lm(formula = EO_Foam_Sway ~ PASE.mean.c * WML_volume.c + age.center +
##     Sex + MoCA.center, data = data)
##
## Residuals:
##     Min      1Q  Median      3Q     Max
## -203.26  -58.69  -18.00   43.53  361.76
##
## Coefficients:
##                            Estimate Std. Error t value Pr(>|t|)
## (Intercept)               1.494e+02  1.413e+01  10.572 6.43e-16 ***
## PASE.mean.c              -6.348e-01  1.803e-01  -3.520 0.000782 ***
## WML_volume.c              1.455e-02  3.884e-03   3.746 0.000376 ***
## age.center                1.684e+00  4.151e+00   0.406 0.686360
## SexM                      3.756e+01  2.774e+01   1.354 0.180253
## MoCA.center               2.086e+00  3.547e+00   0.588 0.558362
## PASE.mean.c:WML_volume.c -2.255e-04  5.384e-05  -4.189 8.38e-05 ***
## ---
## Signif. codes:  0 '***' 0.001 '**' 0.01 '*' 0.05 '.' 0.1 ' ' 1
##
## Residual standard error: 102.4 on 67 degrees of freedom
## Multiple R-squared:  0.3347,    Adjusted R-squared:  0.2751
## F-statistic: 5.617 on 6 and 67 DF,  p-value: 9.071e-05
```

summary(reg.low)

```
## Call:
## lm(formula = EO_Foam_Sway ~ PASE.Low * WML_volume.c + age.center +
##     Sex + MoCA.center, data = data)
##
```

```
## Residuals:
##     Min      1Q  Median      3Q     Max
## -203.26  -58.69  -18.00   43.53  361.76
##
## Coefficients:
##                        Estimate Std. Error t value Pr(>|t|)
## (Intercept)           1.926e+02  1.848e+01  10.419 1.18e-15 ***
## PASE.Low             -6.348e-01  1.803e-01  -3.520 0.000782 ***
## WML_volume.c          2.990e-02  6.608e-03   4.525 2.53e-05 ***
## age.center            1.684e+00  4.151e+00   0.406 0.686360
## SexM                  3.756e+01  2.774e+01   1.354 0.180253
## MoCA.center           2.086e+00  3.547e+00   0.588 0.558362
## PASE.Low:WML_volume.c -2.255e-04  5.384e-05  -4.189 8.38e-05 ***
## ---
## Signif. codes:  0 '***' 0.001 '**' 0.01 '*' 0.05 '.' 0.1 ' ' 1
##
## Residual standard error: 102.4 on 67 degrees of freedom
## Multiple R-squared:  0.3347,    Adjusted R-squared:  0.2751
## F-statistic: 5.617 on 6 and 67 DF,  p-value: 9.071e-05
```

summary(reg.high)

```
## Call:
## lm(formula = EO_Foam_Sway ~ PASE.High * WML_volume.c + age.center +
##     Sex + MoCA.center, data = data)
##
## Residuals:
##     Min      1Q  Median      3Q     Max
## -203.26  -58.69  -18.00   43.53  361.76
##
## Coefficients:
 ##                        Estimate Std. Error t value Pr(>|t|)
## (Intercept)            1.061e+02  1.895e+01   5.601 4.31e-07 ***
## PASE.High             -6.348e-01  1.803e-01  -3.520 0.000782 ***
## WML_volume.c          -8.066e-04  3.658e-03  -0.221 0.826148
## age.center             1.684e+00  4.151e+00   0.406 0.686360
## SexM                   3.756e+01  2.774e+01   1.354 0.180253
## MoCA.center            2.086e+00  3.547e+00   0.588 0.558362
## PASE.High:WML_volume.c -2.255e-04  5.384e-05  -4.189 8.38e-05 ***
```

```
## ---
## Signif. codes:  0 ‘***’ 0.001 ‘**’ 0.01 ‘*’ 0.05 ‘.’ 0.1 ‘ ’ 1
##
## Residual standard error: 102.4 on 67 degrees of freedom
## Multiple R-squared:  0.3347,     Adjusted R-squared:  0.2751
## F-statistic: 5.617 on 6 and 67 DF,  p-value: 9.071e-05
```

```
b0.mid <- round(reg.mean$coeff[1], digits = 3)
PASE.mid <- round(reg.mean$coeff[2], digits = 3)
WML.mid <- round(reg.mean$coeff[3], digits = 6)
interaction.mid <- round(reg.mean$coeff[7], digits = 6)



b0.low <- round(reg.low$coeff[1], digits = 3)
PASE.low <- round(reg.low$coeff[2], digits = 3)
WML.low <- round(reg.low$coeff[3], digits = 6)
interaction.low <- round(reg.low$coeff[7], digits = 6)

b0.high <- round(reg.high$coeff[1], digits = 3)
PASE.high <- round(reg.high$coeff[2], digits = 3)
WML.high <- round(reg.high$coeff[3], digits = 6)
interaction.high <- round(reg.high$coeff[7], digits = 6)
```

```
#Color Figure
ggplot(data, aes(x = WML_volume, y = EO_Foam_Sway)) + geom_point(shape = 1) +
  geom_abline(aes(intercept = b0.mid, slope = WML.mid + PASE.mid*interaction.mid
, color= "Mean PASE")) +
  geom_abline(aes(intercept = b0.low, slope = WML.low + PASE.low * interaction.l
ow, color = "Low PASE")) +
  geom_abline(aes(intercept= b0.high, slope = WML.high + PASE.high*interaction.h
igh, color= "High PASE")) +
  guides(color = guide_legend((title = "PASE Scores"))) + xlab("White Matter Hyp
erintensity Volume") +
  ylab(expression(Foam~Sway~(mm^2))) + theme_bw() + theme(panel.border = element
_blank(), panel.grid.major = element_blank(),
  panel.grid.minor = element_blank(), axis.line = element_line(colour = "black")
)
```

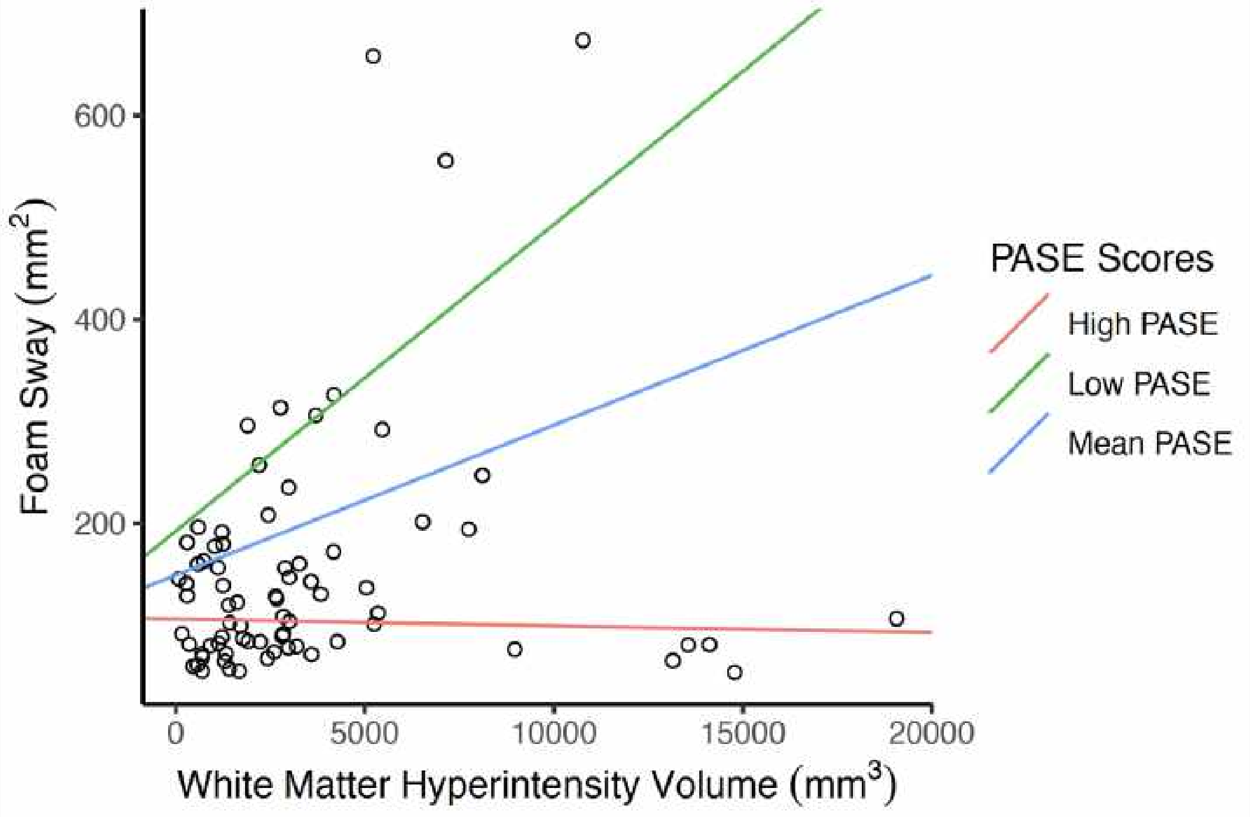


```
#Black and White Figure
```

```
ggplot(data, aes(x = WML_volume, y = EO_Foam_Sway)) + geom_point(shape =
1) +
  geom_abline(aes(intercept = b0.mid, slope = WML.mid +
PASE.mid*interaction.mid, linetype= "Mean PASE")) +
  geom_abline(aes(intercept = b0.low, slope = WML.low + PASE.low *
interaction.low, linetype = "Low PASE")) +
  geom_abline(aes(intercept= b0.high, slope = WML.high +
PASE.high*interaction.high, linetype= "High PASE")) +
  guides(linetype = guide_legend((title = "PASE Scores"))) +
xlab(expression(White~Matter~Hyperintensity~Volume~(mm^3))) +
  ylab (expression(Foam~Sway~(mm^2)))+ theme_bw() + theme(panel.border =
element_blank(), panel.grid.major = element_blank(),
                                            panel.grid.minor =
element_blank(), axis.line = element_line(colour = "black"))
```

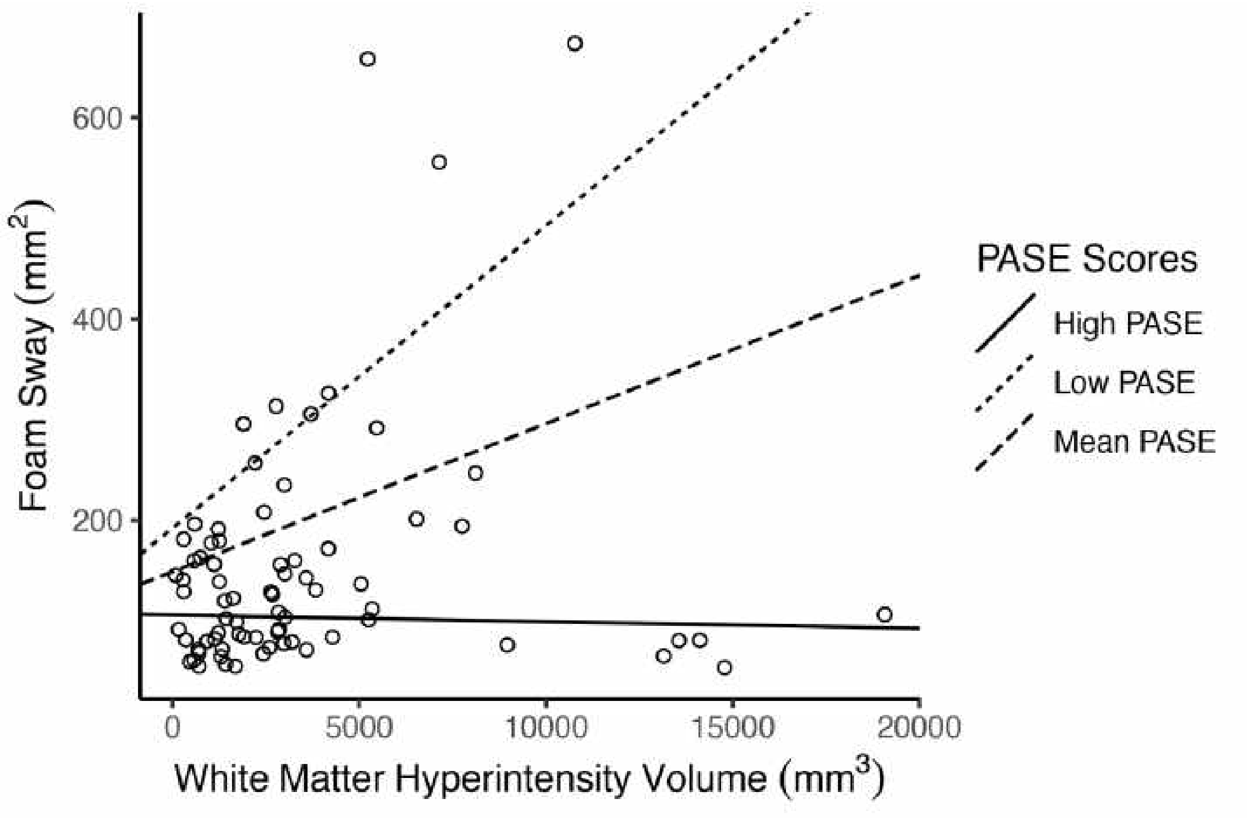
Foam Sway (mm²)
600
400
200
0
5000
10000
15000
20000
White Matter Hyperintensity Volume (mm³)
PASE Scores
High PASE
Low PASE
Mean PASE
